# A narrative review of therapy-induced senescence in cancer: mechanisms, immune interplay, and therapeutic opportunities

**DOI:** 10.1186/s43046-025-00333-8

**Published:** 2025-12-08

**Authors:** Henry Sutanto, Alfan Ahkami, Deasy Fetarayani, Pradana Zaky Romadhon

**Affiliations:** 1 Internal Medicine Study Program, Department of Internal Medicine, Faculty of Medicine, Universitas Airlangga, Surabaya, Indonesia; 2 Department of Internal Medicine, Dr. Soetomo General Academic Hospital, Surabaya, Indonesia; 3 Department of Internal Medicine, Airlangga University Hospital, Surabaya, Indonesia; 4 Division of Allergy and Clinical Immunology, Department of Internal Medicine, Faculty of Medicine, Universitas Airlangga, Surabaya, Indonesia; 5 Division of Hematology and Medical Oncology, Department of Internal Medicine, Faculty of Medicine, Universitas Airlangga, Surabaya, Indonesia

**Keywords:** Therapy-induced senescence, Senescence-associated secretory phenotype, Cancer, Immunology, Oncology

## Abstract

Therapy-induced senescence (TIS) has emerged as a pivotal mechanism in cancer therapy, exerting both tumor-suppressive and tumor-promoting effects. By enforcing stable cell-cycle arrest, TIS prevents proliferation of damaged cancer cells while simultaneously reprogramming the tumor microenvironment (TME) through the senescence-associated secretory phenotype (SASP). This secretome recruits and activates innate and adaptive immune effectors, thereby enhancing immune clearance and sensitizing tumors to immunotherapies such as immune checkpoint inhibitors. However, the chronic persistence of senescent cells and sustained SASP can paradoxically foster immune suppression, angiogenesis, epithelial–mesenchymal transition, and tumor relapse. Preclinical data demonstrate that combining senescence-inducing therapies with immunotherapies produces synergistic effects, particularly when paired with senolytic agents to eliminate residual senescent cells. Early clinical trials, especially those integrating CDK4/6 inhibitors with PD-1/PD-L1 blockade, highlight promising translational opportunities but also underscore critical challenges, including therapy sequencing, SASP heterogeneity, and T-cell suppression. Biomarker development remains essential to monitor senescence dynamics and immune modulation in real time. This review synthesizes mechanistic insights into TIS, its immunological interplay, and emerging therapeutic strategies, advocating for a rational “induce–prime–purge” framework. Leveraging TIS in combination with immunotherapies and senolytics holds the potential to transform refractory tumors into immune-responsive states while minimizing risks of relapse.

## Introduction

Therapy-induced senescence (TIS) has emerged as a critical tumor-suppressive mechanism that contributes to the efficacy of many cancer therapies. Unlike apoptosis, senescence is a state of stable cell cycle arrest in which tumor cells remain metabolically active but cease to proliferate. TIS can be induced by a variety of treatments, including chemotherapy, radiation, and targeted agents that induce non-lethal cellular stress or DNA damage [1, 2]. By halting tumor cell division, TIS provides a potent, though sometimes temporary, barrier to cancer progression [3]. Even in tumors with defective apoptotic pathways, senescence can serve as a fail-safe response to therapy, reinforcing its relevance across various cancer types [4]. Beyond its direct effect on tumor cell proliferation, TIS also exerts a profound influence on the tumor microenvironment (TME) through the senescence-associated secretory phenotype (SASP). SASP encompasses a diverse array of secreted factors, including pro-inflammatory cytokines, chemokines, growth factors, and proteases (Table 1). These molecules can recruit and activate various components of the immune system, including T cells, natural killer (NK) cells, macrophages, and dendritic cells (DCs), thereby facilitating immune-mediated clearance of senescent cells [5]. However, SASP can also have deleterious effects by promoting a pro-tumorigenic environment, angiogenesis, and immune suppression in some contexts [3, 6]. The dual nature of SASP underscores the need for precise modulation when exploiting TIS in cancer therapy. The aim of this review is to provide a comprehensive synthesis of TIS as both a tumor-suppressive mechanism and a therapeutic target in cancer. It examines the molecular pathways through which diverse anticancer treatments trigger senescence, the dual role of SASP in immune activation and suppression, and the capacity of TIS to remodel the TME. The review further highlights how senescence induction can synergize with immunotherapies, particularly immune checkpoint inhibitors (ICIs), while also considering the risks of chronic SASP and senescence escape. Finally, it evaluates emerging strategies such as senolytic agents, alongside the development of predictive biomarkers, to optimize TIS-based interventions.

**Table 1:** SASP molecules and their functions in cancer

SASP Molecule	Type	Primary Function in Cancer
IL-6	Cytokine	Promotes immune cell recruitment (acute), but can drive chronic inflammation, EMT, and tumor progression (chronic)
TNF-α	Cytokine	Can promote inflammation and immune activation; chronic signaling may lead to tissue damage and tumor support
IL-1α/IL-1β	Cytokine	Act as upstream regulators of SASP; amplify expression of other SASP factors and NF-κB signaling
IL-10	Cytokine	Anti-inflammatory; promotes immune suppression and can inhibit T cell and NK cell function
TGF-β	Cytokine	Promotes EMT, fibrosis, and immune suppression; involved in tissue remodeling and therapy resistance
IL-8 (CXCL8)	Chemokine	Attracts neutrophils and supports angiogenesis; enhances tumor cell migration and metastasis
CCL2 (MCP-1)	Chemokine	Recruits monocytes, macrophages, and DCs; may lead to immunosuppression via M2 macrophage polarization
CCL5 (RANTES)	Chemokine	Recruits T cells and NK cells; enhances immune surveillance when acutely induced
CXCL1/CXCL2	Chemokine	Recruit neutrophils and may contribute to inflammation and tumor proliferation
CXCL9/CXCL10	Chemokine	Attract cytotoxic CD8⁺ T cells and NK cells; improve tumor immune infiltration
MMP1/MMP3/MMP9	Proteases	Remodel extracellular matrix (ECM), facilitate tumor invasion and metastasis
GM-CSF	Growth factor	Stimulates myeloid cell differentiation; can enhance antigen presentation but may recruit MDSCs
VEGF	Growth factor	Promotes angiogenesis; supports tumor growth and vascular remodeling
IGFBP-3/IGFBP-7	Binding proteins	Modulate IGF signaling and can reinforce senescence or apoptosis, context-dependent
HMGB1	DAMP	Released from senescent cells; promotes immune activation and antigen presentation
PGE2	Lipid mediator	Contributes to immune evasion and supports tumor-promoting inflammation
PD-L1	Immune checkpoint	Upregulated in some senescent cells; inhibits T cell activity and promotes immune evasion

*CCL2 (MCP-1)* Chemokine (C-C motif) ligand 2, also known as Monocyte Chemoattractant Protein-1, *CCL5 (RANTES)* Chemokine (C-C motif) ligand 5, also known as Regulated on Activation, Normal T Cell Expressed and Secreted, *CXCL1 / CXCL2* Chemokine (C-X-C motif) ligand 1 / 2, *CXCL8 (IL-8)* Chemokine (C-X-C motif) ligand 8, also known as Interleukin 8, *CXCL9* Chemokine (C-X-C motif) ligand 9, *CXCL10* Chemokine (C-X-C motif) ligand 10, *DAMP* Damage-associated Molecular Pattern, *GM-CSF* Granulocyte-Macrophage Colony-Stimulating Factor, *HMGB1* High-Mobility Group Box 1, *IGFBP-3 / IGFBP-7* Insulin-like Growth Factor Binding Protein 3 / 7, *IL-6* Interleukin 6, *IL-1α* Interleukin 1 alpha, *IL-1β* Interleukin 1 beta, *IL-10* Interleukin 10, *MMP1 / MMP3 / MMP9* Matrix Metalloproteinase 1 / 3 / 9, *PD-L1* Programmed Death-Ligand 1, *PGE2* Prostaglandin E2, *TGF-β* Transforming Growth Factor beta, *TNF-α* Tumor Necrosis Factor alpha, *VEGF* Vascular Endothelial Growth Factor

## Mechanisms of Therapy-Induced senescence (TIS) in cancer

### Genotoxic stress

**Fig. 1:**
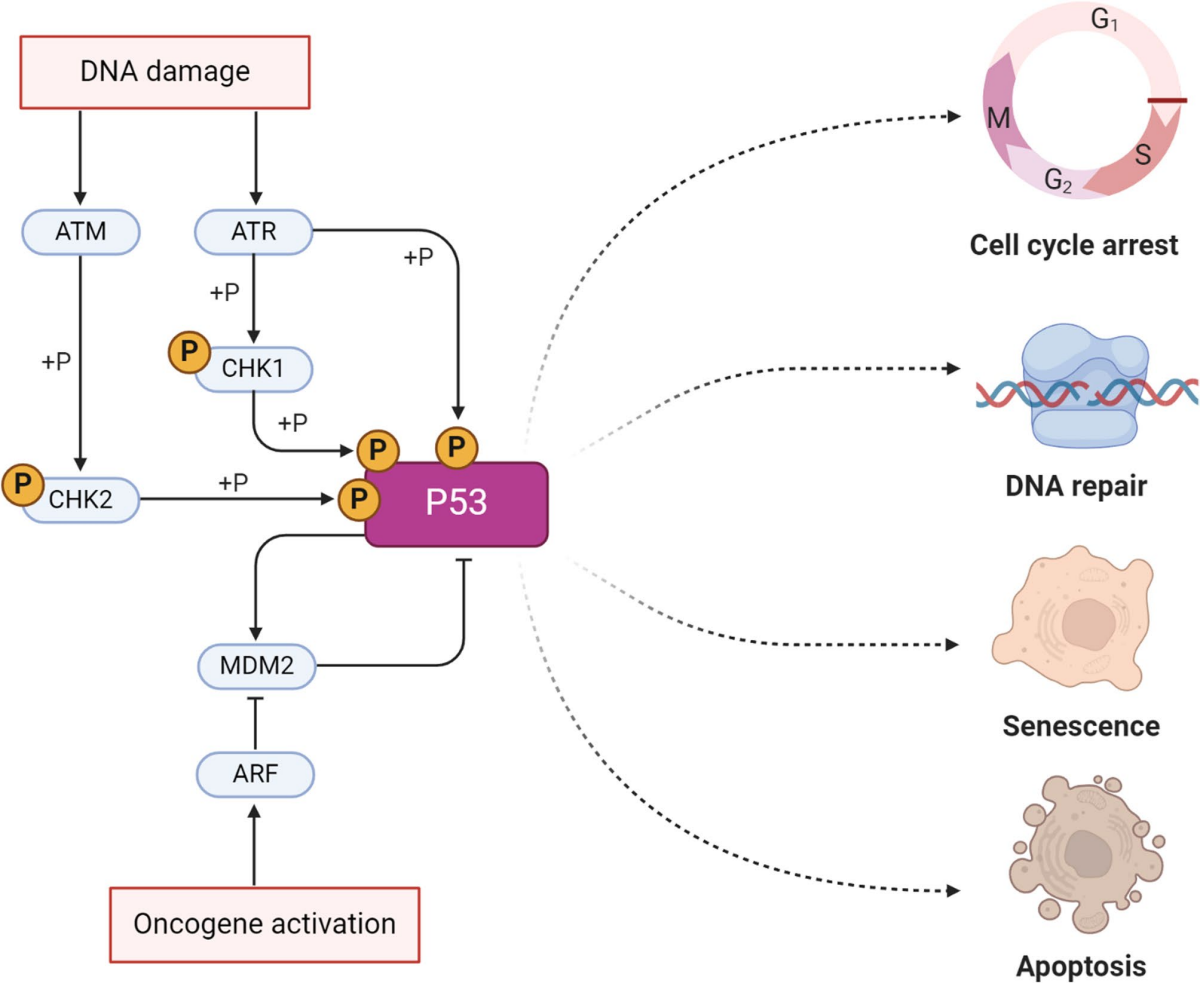
p53-mediated cellular outcomes in response to DNA damage and oncogene activation. DNA damage activates the kinases ATM and ATR, which phosphorylate downstream checkpoint kinases CHK2 and CHK1, respectively. These kinases, in turn, phosphorylate and stabilize p53, preventing its degradation. Activated p53 induces a transcriptional program leading to cell cycle arrest, DNA repair, senescence, or apoptosis, depending on the severity of damage and cellular context. Meanwhile, oncogene activation stimulates ARF, which inhibits MDM2, a negative regulator of p53, thus enhancing p53 stability. This tightly regulated pathway is crucial for preventing the propagation of damaged DNA and tumorigenesis. (ATM = Ataxia Telangiectasia Mutated; ATR = ATM and Rad3-Related; CHK1/CHK2 = Checkpoint Kinase 1 and 2; p53 = Tumor suppressor protein 53; ARF = Alternate Reading Frame protein; MDM2 = Mouse Double Minute 2 homolog; +P = Phosphorylation)

TIS triggered by genotoxic stress represents a well-characterized mechanism by which cancer cells undergo irreversible growth arrest in response to DNA-damaging agents. These agents include chemotherapy (e.g., doxorubicin and etoposide), radiation, and targeted therapies, which cause direct or indirect damage to the DNA, activating a cascade of signaling events aimed at halting cell cycle progression and preserving genomic integrity [7, 8]. Central to this process is the DNA damage response (DDR), a tightly regulated signaling network that detects DNA lesions and coordinates cell cycle arrest, repair, or senescence (Table 2) [8]. Genotoxic stress is primarily sensed by ataxia telangiectasia mutated (ATM) and ataxia telangiectasia and Rad3-related (ATR) kinases. These PI3K-like kinases are rapidly recruited to DNA double-strand breaks (DSBs) and single-strand DNA (ssDNA), respectively. ATM is activated at DSBs through autophosphorylation and interaction with the MRE11-RAD50-NBS1 (MRN) complex, which facilitates ATM localization to damage sites. Once activated, ATM phosphorylates downstream effectors such as checkpoint kinase 2 (CHK2) and p53, initiating a broad transcriptional program [2, 9, 10]. ATR, in contrast, responds to replication stress and ssDNA regions coated by replication protein A (RPA) and activates CHK1 via its co-activator TopBP1. Upon activation, p53 is stabilized and accumulates in the nucleus, where it transcriptionally activates several target genes, most notably *CDKN1A* (p21^Cip1/Waf1^), a potent inhibitor of cyclin-dependent kinases (CDKs) [4, 9, 11]. p21 binds and inhibits CDK2 and CDK4/6, preventing phosphorylation of the retinoblastoma protein (Rb). Hypophosphorylated Rb sequesters E2F transcription factors, halting G1/S transition and enforcing cell cycle arrest (Fig. 1). Persistent activation of this pathway leads to an irreversible state of senescence rather than transient arrest [4, 9, 12].

**Table 2:** Mechanisms of therapy-induced senescence

Mechanism	Key Inducers	Core Molecules Involved	Signaling Pathways/Cascades
DNA Damage Response (DDR)	Chemotherapy (e.g., doxorubicin), ionizing radiation	ATM, ATR, CHK1, CHK2, γH2AX, p53, p21, Rb	DNA double-strand breaks → ATM/ATR → CHK1/CHK2 → p53 stabilization → p21 induction → CDK inhibition → Rb activation
CDK4/6 Inhibition	Palbociclib, ribociclib, abemaciclib	CDK4/6, Cyclin D, Rb, E2F, p16, p21	CDK4/6 inhibition → Rb hypophosphorylation → E2F repression → G1 arrest and senescence
Oncogene-Induced Stress	Ras, Myc overexpression	p16^INK4a^, ARF, p53, Rb	Oncogenic signaling → ARF activation → p53 → p21 or p16 → Rb pathway enforcement
Oxidative Stress	ROS, mitochondrial dysfunction	ROS, p53, p38 MAPK, JNK, NF-κB	ROS accumulation → DNA damage & p38/JNK activation → p53/p21 pathway + NF-κB-mediated SASP
Aurora Kinase A (AURKA) Inhibition	MLN8237 (alisertib), other mitotic kinase inhibitors	AURKA, spindle assembly proteins, DNA damage markers (γH2AX), p53, p21	Mitotic slippage/polyploidy → DDR activation → p53 → p21 → senescence
Telomere Dysfunction	Telomere shortening, replication stress	Shelterin complex, DDR proteins (ATM, γH2AX), p53, p21	Dysfunctional telomeres → DDR → p53 pathway → cell cycle arrest
Epigenetic Disruption	EZH2 inhibitors, HDAC inhibitors	EZH2, H3K27me3, SAHF, p16, p21	Chromatin remodeling → derepression of senescence genes → stable growth arrest
Endoplasmic Reticulum Stress	Hypoxia, proteotoxic stress	PERK, ATF4, CHOP, p21	Unfolded Protein Response (UPR) → PERK/ATF4 → p21 induction → cell cycle arrest
Mitochondrial Dysfunction-Associated Senescence (MiDAS)	Mitochondrial DNA damage, metabolic stress	AMPK, p53, p21, ROS	Metabolic dysfunction → AMPK activation + ROS → p53 stabilization → p21-mediated arrest
Cytokine Reinforcement (Autocrine/Paracrine)	IL-1α, IL-6, TNF-α from SASP	IL-1R, NF-κB, C/EBPβ	Cytokine signaling → NF-κB/C/EBPβ → reinforcement of SASP and senescence loop

Simultaneously, DNA damage activates other pathways contributing to senescence. For example, ATM and ATR also phosphorylate histone variant H2AX on Ser139 (γH2AX), which marks damaged chromatin and recruits DNA repair proteins, forming DNA damage foci (DDF). Persistent DDF are a hallmark of senescent cells, often co-localizing with proteins such as 53BP1 and MDC1. The failure to repair severe or chronic DNA lesions maintains sustained DDR signaling and stabilizes the senescent phenotype [13–15]. Beyond p53-p21-Rb signaling, p16^INK4a^ (encoded by *CDKN2A*) can be independently upregulated in response to genotoxic stress, particularly in p53-deficient contexts. p16 inhibits CDK4/6 activity, reinforcing Rb activation and acting as a parallel gatekeeper of senescence. This redundancy ensures that senescence can be induced even in tumors with disrupted p53 function [16, 17]. Finally, the culmination of these molecular events not only results in cell cycle arrest but also initiates the SASP via NF-κB and C/EBPβ activation, which are often induced downstream of DDR and ROS signaling. SASP factors such as interleukin (IL)−6, IL-8, CCL2, and matrix metalloproteinases (MMPs) reshape the TME by promoting immune cell recruitment and, paradoxically, inflammatory signals that can promote tumorigenesis if not properly regulated [18, 19].

### Oxidative stress

While genotoxic stress represents the canonical trigger of senescence through DNA damage and checkpoint activation, oxidative stress provides an additional and often interconnected pathway that reinforces the DNA damage response and amplifies senescence signaling (Table 2). Oxidative stress in the context of TIS arises from elevated levels of ROS—highly reactive molecules such as superoxide (O₂⁻), hydrogen peroxide (H₂O₂), and hydroxyl radicals (·OH)—which cause oxidative damage to DNA, proteins, and lipids. Many cancer therapies, including radiation, certain chemotherapies, and targeted inhibitors, generate ROS either directly or indirectly, leading to persistent cellular stress and senescence initiation [20, 21]. At the cellular level, ROS overproduction is largely driven by mitochondrial dysfunction. Chemotherapy agents like doxorubicin and cisplatin can damage mitochondrial DNA and impair components of the electron transport chain (ETC), leading to electron leakage and conversion of oxygen into superoxide [22, 23]. This mitochondrial ROS generation activates the DDR cascade, including ATM/ATR kinases, γH2AX, and p53 signaling, thereby reinforcing senescence via the p53–p21^Cip1/Waf1^–Rb pathway. ROS can also damage nuclear DNA directly, creating oxidative lesions like 8-oxoguanine, which are recognized by the base excision repair (BER) pathway but often persist under high oxidative conditions, sustaining the DDR [24]. In parallel, oxidative stress activates multiple signaling pathways that converge on senescence regulators. One critical mediator is p38 MAPK, a stress-activated protein kinase that is strongly induced by ROS. Activated p38 phosphorylates and stabilizes p53, leading to increased transcription of *CDKN1A* (p21) [25]. It also directly influences C/EBPβ and NF-κB, inducing SASP. p38 MAPK signaling promotes the production of SASP components such as IL-6, IL-8, and MMPs, which reinforce growth arrest in an autocrine manner and modulate the TME in a paracrine fashion [26, 27].

**Fig. 2:**
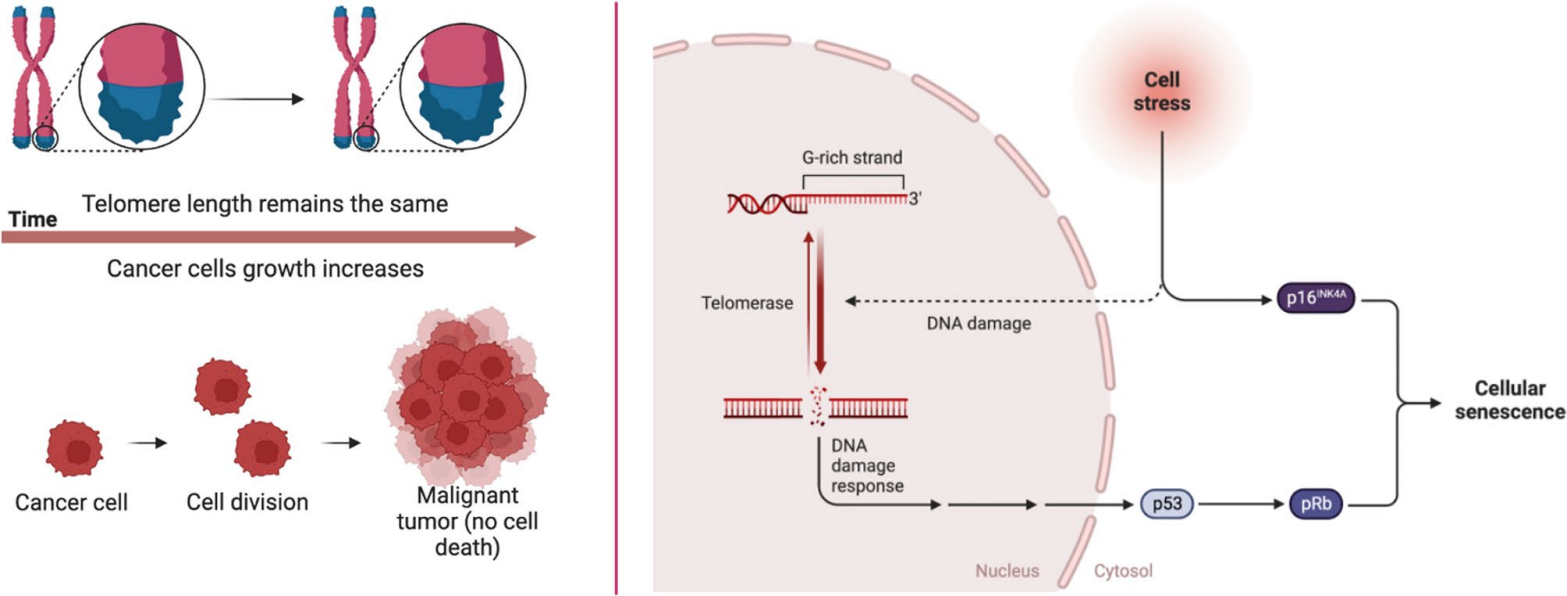
Telomere maintenance and the role of telomerase in cancer cell immortality and stress-induced senescence. In normal cells, telomeres shorten with each cell division, eventually leading to replicative senescence. However, in cancer cells, the enzyme telomerase maintains telomere length, allowing indefinite cell division and tumor growth without cell death. On the left, the schematic shows how persistent telomere length allows cancer cells to proliferate and form malignant tumors. On the right, telomerase activity in the nucleus synthesizes telomeric DNA to compensate for shortening, but DNA damage or critically short telomeres trigger a DNA damage response. Cellular stress induces the activation of p16^INK4A^, which inhibits CDK activity and prevents phosphorylation of pRb, leading to cell cycle arrest. In parallel, DNA damage activates the p53 pathway, further contributing to the establishment of cellular senescence

Another major ROS-responsive transcription factor is nuclear factor erythroid 2–related factor 2 (NRF2). Under normal conditions, NRF2 is bound by KEAP1 and targeted for proteasomal degradation. Upon oxidative stress, KEAP1 is oxidized and releases NRF2, allowing its translocation into the nucleus where it activates antioxidant response genes such as NAD(P)H quinone dehydrogenase 1 (*NQO1*), glutamate-cysteine ligase catalytic (*GCLC*), and heme oxygenase 1 (*HO-1*). In senescent cells, however, persistent ROS overwhelms this protective response, and chronic NRF2 activation can paradoxically contribute to tumor progression by supporting cell survival and SASP production [28–30]. ROS also stimulates NF-κB, a master regulator of inflammation and SASP. Oxidative stress leads to phosphorylation of IκB kinase (IKK), which degrades IκBα and releases NF-κB (p65/p50) dimers into the nucleus. NF-κB then drives the expression of a broad range of inflammatory genes such as TNF-α, IL-1β, and CXCL1, contributing to the pro-inflammatory SASP and reinforcing senescence cell-autonomously and non-cell-autonomously [5, 31, 32]. Moreover, oxidative stress can affect telomeres, which are particularly susceptible to ROS-induced damage. Telomeric DNA lacks robust repair mechanisms, and ROS-induced damage in these regions can lead to telomere-associated foci (TAF), even in the absence of significant telomere shortening. These persistent TAF activate a localized DDR that sustains senescence signaling independently of overall genomic integrity (Fig. 2) [33]. Finally, ROS and oxidative stress are not only inducers of TIS but also consequences of the senescence program itself. Senescent cells display elevated mitochondrial mass and metabolic reprogramming, resulting in sustained ROS production. This feedback loop helps maintain the senescent state and propagates oxidative signals to neighboring cells, a phenomenon referred to as the bystander effect, further enhancing tumor suppression or, paradoxically, tumor promotion depending on the context [31, 34].

### CDK4/6-mediated inhibition

**Fig. 3:**
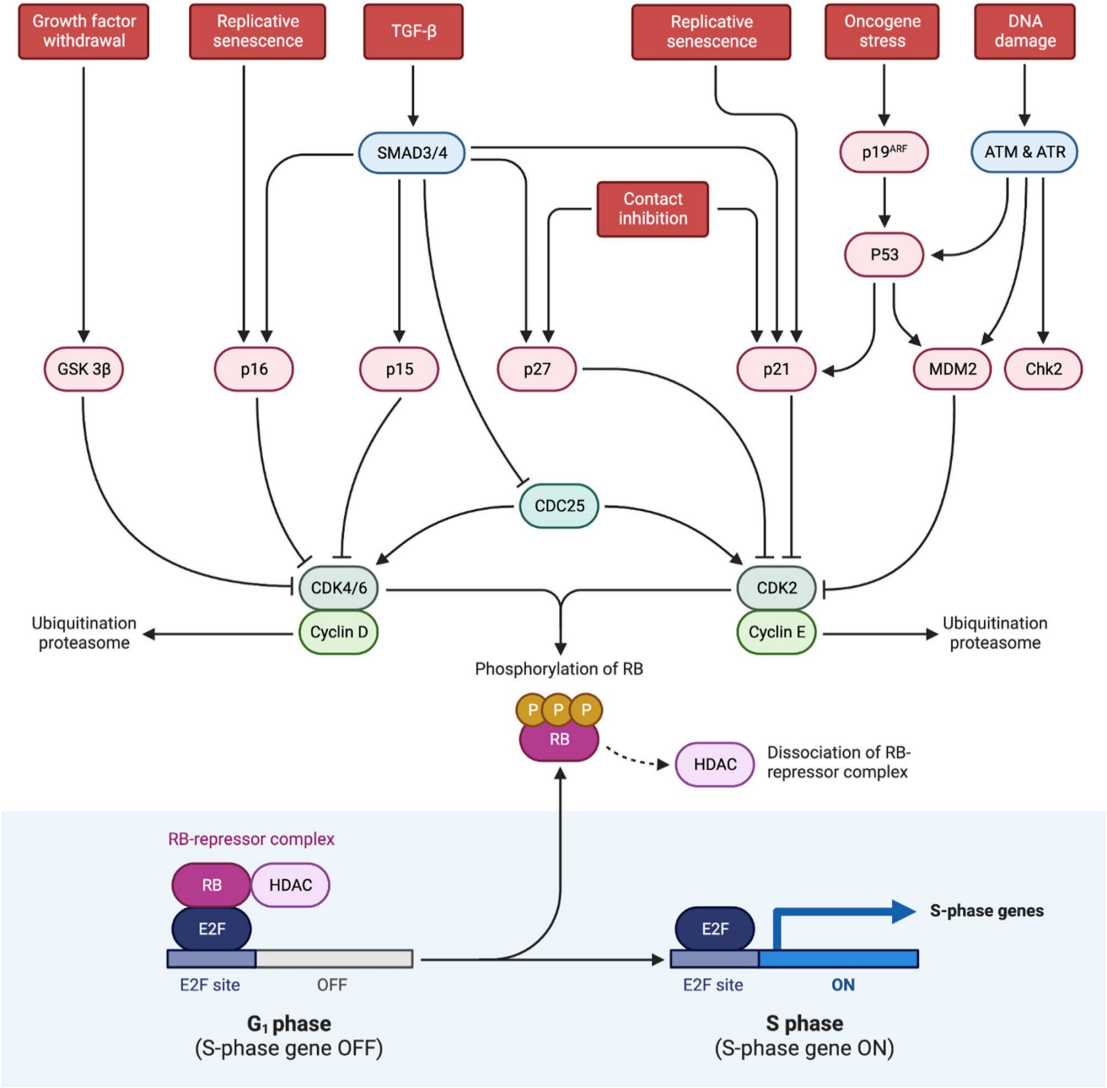
Regulation of the G1/S cell cycle transition by cyclin-dependent kinase inhibitors and stress signals. Activation of CDK4/6-Cyclin D and CDK2-Cyclin E complexes leads to phosphorylation of the retinoblastoma protein (RB), resulting in the dissociation of the RB-E2F repressor complex and activation of S-phase genes. Various cellular signals, including growth factor withdrawal, replicative senescence, TGF-β signaling, contact inhibition, oncogenic stress, and DNA damage, converge to inhibit CDK activity through the upregulation of CKIs such as p15, p16, p21, and p27. Inhibition of CDKs prevents RB phosphorylation, maintaining E2F in an inactive state and halting progression into S phase. The DNA damage response, mediated by ATM/ATR and downstream effectors such as p53 and Chk2, enhances this checkpoint by upregulating p21 and inhibiting CDC25 phosphatases. Ubiquitination and proteasomal degradation also regulate CDK activity by targeting cyclins and CKIs. (ATM = Ataxia Telangiectasia Mutated; ATR = ATM and Rad3-related; CDK = Cyclin-Dependent Kinase; CDC25 = Cell Division Cycle 25 phosphatase; Chk2 = Checkpoint Kinase 2; E2F = E2 promoter-binding factor; GSK 3β = Glycogen Synthase Kinase 3 beta; HDAC = Histone Deacetylase; MDM2 = Mouse Double Minute 2 homolog; p19^ARF^ = Alternate Reading Frame product of the CDKN2A locus; pRB or RB = Retinoblastoma protein; SMAD3/4 = SMAD Family Member 3 and 4; TGF-β = Transforming Growth Factor Beta)

Beyond damage and redox imbalance, targeted inhibition of cell cycle kinases—particularly CDK4/6—can mimic senescence induction by directly enforcing Rb-dependent arrest, providing a therapeutically controllable form of TIS. The targeted inhibition of CDK4/6 represents a precise and clinically relevant strategy to induce TIS (Table 2), particularly in hormone receptor–positive breast cancers and other solid tumors [35, 36]. CDK4 and CDK6 are essential for the transition from the G1 phase to the S phase of the cell cycle. Their activity is regulated through binding to D-type cyclins (Cyclin D1, D2, and D3), which promotes the phosphorylation of the Rb. In its phosphorylated form, Rb releases E2F transcription factors, enabling the transcription of genes required for DNA replication and S-phase entry. Inhibiting CDK4/6 using agents like palbociclib, abemaciclib, or ribociclib disrupts this critical axis. CDK4/6 inhibitors prevent Rb phosphorylation, resulting in the continued sequestration of E2Fs by hypophosphorylated Rb. This effectively halts the cell cycle in early G1 phase and imposes a stable growth arrest, a hallmark of cellular senescence (Fig. 3) [36, 37]. Unlike transient arrest, which may be reversible, prolonged CDK4/6 inhibition triggers senescence by stabilizing cell cycle inhibitors and initiating a downstream transcriptional reprogramming characteristic of senescent cells. A critical mediator of this process is p21^Cip1/Waf1^, encoded by *CDKN1A*. CDK4/6 inhibition, either directly or via upstream DNA damage and stress signals, leads to activation of p53, which transcriptionally upregulates p21. p21 inhibits not only CDK2 but also reinforces CDK4/6 inhibition, promoting a feedback loop that deepens the arrest [38]. Similarly, p16^INK4a^ (CDKN2A) is often upregulated during prolonged CDK4/6 inhibition, functioning independently of p53 to inhibit CDK4/6 directly. Together, p21 and p16 lock the cell cycle machinery in a repressive state by maintaining Rb in its active, hypophosphorylated form [39]. This senescent phenotype is reinforced by chromatin remodeling and epigenetic alterations. CDK4/6 inhibition promotes the formation of senescence-associated heterochromatin foci (SAHF)—dense regions of facultative heterochromatin that silence E2F target genes, further preventing cell cycle re-entry [40]. SAHF formation involves deposition of H3K9me3 histone marks, recruitment of heterochromatin protein 1 (HP1α), and downregulation of histone variant H2A.Z, which together contribute to the transcriptional silencing of proliferation-related genes [41].

Importantly, CDK4/6-induced senescence is not a purely cell-autonomous process. Senescent cells develop a SASP composed of pro-inflammatory cytokines (e.g., IL-6, IL-8), chemokines (CCL2, CXCL10), matrix-remodeling enzymes (MMP1, MMP3), and growth factors (VEGF, GM-CSF). This secretory profile is largely regulated by transcription factors NF-κB and C/EBPβ, which are activated in part by persistent DDR and stress signaling even in the absence of extensive DNA damage [5, 18, 19]. Notably, the composition of SASP induced by CDK4/6 inhibitors is distinct from that triggered by genotoxic therapies, often skewing toward an immune-activating phenotype rather than a pro-tumorigenic one [42]. Emerging data suggest that CDK4/6 inhibition may also modulate antigen presentation machinery. By inducing senescence, these inhibitors can increase expression of MHC class I molecules, NKG2D ligands, and interferon-stimulated genes (ISGs), potentially enhancing immune recognition by cytotoxic T lymphocytes (CTLs) and NK cells [43–45].

### Aurora kinase A-mediated inhibition

Similar to CDK4/6 blockade, inhibition of mitotic regulators such as Aurora kinase A (AURKA) imposes mitotic stress and chromosomal instability, culminating in a senescence-like arrest accompanied by inflammatory signaling. AURKA is a serine/threonine kinase critical for mitotic progression. It governs key mitotic processes, including centrosome maturation, bipolar spindle formation, and chromosome alignment during metaphase [46]. Overexpression or hyperactivation of AURKA is frequently observed in a range of cancers, including breast, prostate, and melanoma, where it promotes genomic instability, unchecked proliferation, and resistance to therapy [46, 47]. Inhibition of AURKA has therefore emerged as a promising anti-cancer strategy, not only for inducing mitotic arrest and apoptosis but also for triggering TIS in tumor cells [48, 49]. Inhibition of AURKA disrupts proper mitotic spindle assembly, leading to defective chromosome segregation, polyploidy, and mitotic slippage, where cells exit mitosis abnormally without completing cytokinesis. These aberrant mitotic events result in aneuploidy, supernumerary centrosomes, and accumulation of cytosolic chromatin fragments, which collectively act as potent triggers of cellular stress responses. One of the immediate consequences is the activation of the DDR pathway, even in the absence of exogenous genotoxic agents. DNA damage sensors such as ATM and ATR become activated and phosphorylate key substrates including CHK1/CHK2, γH2AX, and p53, initiating the transcriptional program that enforces senescence [50, 51]. p53, once stabilized by post-translational modifications (e.g., phosphorylation at Ser15), induces the expression of *CDKN1A* (p21^Cip1/Waf1^), a cyclin-dependent kinase inhibitor that suppresses CDK2 activity. This leads to hypophosphorylation of the Rb, resulting in Rb-mediated repression of E2F transcription factors, thereby halting cell cycle progression in G1 phase [52]. This p53–p21–Rb axis is central to the senescence phenotype observed after AURKA inhibition.

Interestingly, in many tumor types with p53 mutations, AURKA inhibition can still induce senescence via p16^INK4a^ (*CDKN2A*), another CDK inhibitor. p16 acts upstream of CDK4/6 and reinforces Rb activation, thereby enforcing cell cycle arrest in a p53-independent manner. This redundancy in senescence induction pathways ensures that AURKA inhibition remains effective even in tumors with defective p53 or DNA repair machinery [52, 53]. Moreover, AURKA inhibition has been shown to elicit a SASP, which comprises a broad array of cytokines (e.g., IL-6, IL-8, CCL5), growth factors, and proteases. This is driven by activation of the NF-κB and C/EBPβ transcriptional programs, which are stimulated by persistent DDR signaling and chromatin stress [5, 54]. One study found that AURKA inhibitors induced CCL5 expression in melanoma models, leading to the recruitment of tumor-infiltrating lymphocytes (TILs) and remodeling of the immune microenvironment [48]. This SASP-driven immunogenicity plays a pivotal role in the therapeutic efficacy of AURKA inhibitors. Senescent tumor cells, via their SASP, upregulate antigen-presenting machinery (e.g., major histocompatibility complex class I [MHC class I]) and ligands for activating immune receptors (e.g., NKG2D), which can stimulate immune effector cells such as CD8⁺ T cells and NK cells [55, 56]. This results in immune-mediated clearance of senescent cells and tumor regression, especially when AURKA inhibitors are combined with ICIs or T cell co-stimulation strategies [57, 58]. In detail, a study investigated the effects of AURKA inhibitor MLN8237. It reported that MLN8237 significantly upregulated PD-L1 expression in a time- and dose-dependent manner, likely through STAT3 phosphorylation. AURKA knockdown similarly increased PD-L1 expression. In mouse tumor models, MLN8237 reduced CD3 + and CD8^+^ T cell infiltration, suggesting an immunosuppressive TME. However, combining MLN8237 with an anti-PD-L1 antibody reversed this effect, leading to enhanced T cell infiltration and greater tumor shrinkage. These findings suggest that MLN8237, while promoting immune evasion via PD-L1 upregulation, may enhance the efficacy of immunotherapy when used in combination with PD-L1 blockade, offering a potential strategy to overcome resistance in targeted cancer therapy [57]. Another study evaluated the effects of Aurora A inhibition via alisertib on apoptosis, DNA damage, and immunogenic cell death (ICD) in HPV-positive cancer cells, as well as its therapeutic potential alone and in combination with ICIs in murine models. Alisertib induced apoptosis, DNA damage, and ICD in vitro, and notably caused selective depletion of immunosuppressive myeloid-derived suppressor cells (MDSCs). In vivo, combining alisertib with anti-CTLA-4 significantly improved survival, enhanced CD8^+^ T cell infiltration, and reduced MDSC levels in the TME—effects not observed with alisertib or ICIs alone. These findings highlight a synergistic interaction between Aurora A inhibition and CTLA-4 blockade [58]. Yet, senescence is not limited to canonical p53–Rb signaling. When these tumor suppressor pathways are compromised, cells engage alternative stress responses and epigenetic programs to sustain a senescent phenotype—mechanisms discussed in the following section.

### Other non-canonical mechanisms

While the canonical p53–p21–Rb axis is a primary enforcer of TIS in many cancer cells, senescence can still occur in the absence of functional TP53 and RB1, indicating the presence of alternative, compensatory signaling cascades. These alternative pathways involve both transcriptional and epigenetic regulatory mechanisms that engage CDK inhibitors, stress kinases, and pro-inflammatory signaling, collectively leading to a senescent phenotype even in p53-incompetent contexts [39, 59]. One of the central alternative mediators in p53-deficient cells is p16^INK4a^ (*CDKN2A*), which is frequently upregulated in response to oncogenic stress and various anticancer treatments. p16 acts independently of p53 by directly inhibiting CDK4 and CDK6, preventing phosphorylation of Rb and enforcing G1 arrest [39]. In Rb-deficient cells, however, the role of p16 must be compensated by other mechanisms, given that Rb is the main effector of CDK4/6 inhibition. In such cases, senescence can be sustained through Rb family members such as p107 (RBL1) and p130 (RBL2), which can substitute for Rb in repressing E2F transcription factors and halting the cell cycle [59, 60]. In cells lacking both p53 and Rb1, a non-canonical senescence program can still be initiated via persistent DDR signaling. In these cells, DNA damage from chemotherapy, radiation, or targeted therapies activates ATM/ATR kinases, which phosphorylate histone H2AX (γH2AX), CHK2, and 53BP1, forming DDF. These foci persist due to the inability of mutant cells to efficiently repair damage, leading to continued activation of stress-activated kinases such as p38 MAPK and JNK [13]. p38 MAPK, in particular, plays a central role in establishing senescence in the absence of p53. Activation of p38 in response to oxidative stress or unresolved DDR enhances transcriptional activity of C/EBPβ and NF-κB, which together drive the SASP. This inflammatory secretome reinforces growth arrest in an autocrine/paracrine fashion and recruits immune cells to eliminate damaged cells. Notably, this process can occur without classical p53-mediated gene expression, suggesting that SASP-associated transcription factors can compensate for the loss of tumor suppressors by enforcing senescence through inflammatory reprogramming [61, 62]. Further supporting this, studies in MYC-driven tumors—which often harbor p53 and Rb1 deletions—have shown that treatment with CDK inhibitors (e.g., CDK4/6 or CDK9 inhibitors) can induce a non-canonical senescence-like state characterized by SASP induction, immune activation, and durable growth arrest. Even without functional p53 and Rb, these tumors exhibit upregulation of cell cycle repressors, such as p27^Kip1^ (*CDKN1B*), and increased expression of ISGs, facilitating immune recognition [63, 64].

Another important adaptation involves epigenetic reprogramming. In the absence of p53/Rb signaling, senescence can be enforced by chromatin remodeling through histone methylation (e.g., H3K9me3, H3K27me3) and recruitment of transcriptional repressors such as heterochromatin protein 1 alpha (HP1α) and enhancer of zeste homolog 2 (EZH2). A study investigated why TIS (via MEK inhibition with trametinib and CDK4/6 inhibition with palbociclib) elicits strong NK cell–mediated tumor control in KRAS-mutant lung cancer but not in pancreatic ductal adenocarcinoma (PDAC). In PDAC, the pancreatic TME suppressed SASP and NK cell immunity through EZH2-mediated epigenetic repression of key proinflammatory SASP factors and NK cell–activating cytokines (e.g., IL-15, IL-18) and chemokines. Genetic or pharmacological inhibition of EZH2 restored SASP activity, enhanced NK cell recruitment, and enabled tumor cytotoxicity in murine and human PDAC models. Combining EZH2 blockade with TIS even produced complete tumor regressions in some PDAC-bearing mice, an effect dependent on NK cells and SASP signaling. Transcriptomic data from human PDAC samples supported this mechanism, linking EZH2 repression signatures with improved NK infiltration and SASP induction [65, 66]. Lastly, emerging evidence shows that cells lacking TP53 and RB1 can enter a polyploidy-associated senescence state. Following mitotic slippage or cytokinesis failure—often triggered by Aurora kinase or microtubule inhibitors—cells become polyploid and undergo metabolic and transcriptomic reprogramming characteristic of senescence, including increased mitochondrial ROS, lipofuscin accumulation, and SASP secretion [67, 68]. Regardless of the initiating trigger, senescent tumor cells profoundly remodel their surroundings through SASP secretion. The next section explores how these secreted mediators recruit and activate innate and adaptive immune effectors within the TME.

### Recruitment of immune effector cells via TIS-induced cytokines

Senescent cells produce a variety of chemokines, including CCL2 (MCP-1), CCL5 (RANTES), CXCL1, CXCL2, and CXCL10, which attract immune cells such as NK cells, CD8^+^ CTLs, DCs, and macrophages [69, 70]. For instance, in melanoma models, therapy-induced senescence via AURKA or CDK4/6 inhibition led to the upregulation of CCL5, a chemokine known to recruit T cells and other leukocytes to the tumor site. This recruitment was essential for effective tumor control, as depletion of CCL5 or immune effector cells diminished the therapeutic benefit of AURKA inhibition [48]. In addition to chemokines, TIS triggers the secretion of cytokines such as IL-6, IL-8, IL-1α, and TNF-α, which act not only as inflammatory signals but also as immunomodulatory agents that activate innate and adaptive immune cells [4]. IL-6, for example, enhances antigen presentation by increasing MHC class I expression on tumor cells and promotes the differentiation of Th17 cells, which are involved in tumor surveillance. Meanwhile, IL-8 (CXCL8) recruits neutrophils and NK cells and supports their activation within the TME [5, 71]. Importantly, the effectiveness of TIS-induced immune recruitment is context-dependent. In immunologically “cold” tumors, such as pancreatic cancer, senescence alone may be insufficient to stimulate immune infiltration due to the suppressive TME. However, modulation of SASP composition or combination with agents that alter the epigenetic regulation of SASP genes (e.g., EZH2 inhibitors) can restore cytokine production and enhance NK cell recruitment and cytotoxicity, as demonstrated in models of KRAS-driven pancreatic cancer [72, 73]. Furthermore, TIS can upregulate stress-induced ligands such as MICA/B and ULBPs, which are recognized by the NKG2D receptor on NK cells and γδ T cells. These ligands promote the direct killing of senescent tumor cells, providing an important mechanism for immune-mediated clearance of senescent populations. The interplay between SASP factors and these ligands amplifies the immune visibility of senescent cells, particularly when combined with ICIs or adoptive immune cell therapies [74, 75].

## SASP-mediated immune activation vs. suppression

TIS has long been recognized as a powerful tumor-suppressive mechanism. By halting the proliferation of damaged or stressed tumor cells, TIS prevents the propagation of potentially malignant clones and promotes a state of durable growth arrest. However, accumulating evidence has revealed a Janus-faced nature of TIS, where the same processes that initially suppress tumor progression can paradoxically contribute to tumor relapse, progression, and metastasis [6]. A major contributor to this paradox is the SASP, which while immunostimulatory in acute contexts, can become chronic and immunosuppressive if senescent cells persist [76]. Persistent SASP factors, such as IL-6, IL-8, MMPs, and TGF-β, can promote a pro-inflammatory and tumor-promoting microenvironment, enhance epithelial-to-mesenchymal transition (EMT), stimulate angiogenesis, and even support the proliferation of nearby non-senescent tumor cells through paracrine signaling [4, 77–79]. SASP secretion is primarily regulated at the transcriptional level by NF-κB and C/EBPβ, both of which are activated downstream of persistent DDR signaling [80]. Upon genotoxic stress, sensors such as ATM and ATR phosphorylate CHK2 and γH2AX, which recruit 53BP1 and MDC1 to damage foci, stabilizing the DDR. Chronic DDR leads to the stabilization and nuclear translocation of NF-κB (particularly p65/RelA) via the IKK complex, which phosphorylates and degrades IκBα, an NF-κB inhibitor. Activated NF-κB then drives the expression of SASP genes, including IL-6, IL-8, and MMPs, promoting both inflammation and extracellular matrix (ECM) remodeling [10, 81, 82].

The pro-inflammatory milieu generated by SASP can have tumor-promoting consequences. Chronic secretion of IL-6 and IL-8, for example, activates JAK/STAT3 and MAPK/ERK signaling pathways in surrounding tumor cells, enhancing proliferation, angiogenesis, and EMT [83]. EMT is a process by which epithelial cancer cells acquire mesenchymal features, increasing their motility, invasiveness, and resistance to therapy. SASP-induced TGF-β further promotes EMT and contributes to the creation of a fibrotic and immunosuppressive tumor stroma [84]. SASP also remodels the immune landscape in a context-dependent manner. While early SASP factors such as CCL2 and CXCL10 recruit cytotoxic immune cells (e.g., NK cells, CD8⁺ T cells), prolonged SASP exposure can lead to the recruitment and polarization of immunosuppressive cell types, including M2 macrophages, MDSCs, and regulatory T cells (Tregs). These cells suppress cytotoxic immune responses and contribute to immune evasion [85, 86]. For instance, in prostate cancer models, SASP induced by CDK inhibitors enhanced NK cell recruitment but also polarized macrophages to an M2-like, tumor-supportive phenotype, thereby limiting overall anti-tumor efficacy [5, 87]. Furthermore, SASP-induced MMPs degrade components of the ECM, facilitating tumor cell invasion and metastasis. This proteolytic remodeling also releases ECM-bound growth factors such as VEGF, which enhances angiogenesis and supports tumor growth. Additionally, autocrine SASP signaling can reinforce the senescence state in a feed-forward loop, while paracrine signaling from senescent stromal or tumor cells can induce senescence in adjacent cells, contributing to tissue dysfunction and heterogeneity within the TME [6, 88]. In cases where senescent cells persist due to inefficient immune clearance, the TME becomes increasingly inflamed, fibrotic, and immunosuppressed—an ideal setting for tumor relapse and progression. This has been observed in preclinical models of melanoma and pancreatic cancer, where SASP-related CCL5 secretion was found to recruit T cells only in the presence of co-stimulatory immunotherapy; otherwise, it had minimal tumor-suppressive effects [89].

## Senescence escape and reversal

Although TIS was initially regarded as a stable and irreversible growth arrest, accumulating evidence reveals that senescent tumor cells can escape this state, re-enter the cell cycle, and contribute to tumor relapse, metastasis, and therapy resistance. This phenomenon—termed senescence escape or reversal—is driven by a complex interplay of epigenetic changes, cell cycle reactivation, immune evasion, and metabolic reprogramming, often occurring under conditions of chronic stress, hypoxia, or incomplete clearance of senescent cells [4, 90, 91]. At the heart of senescence maintenance are the CDK inhibitors p16^INK4a^ and p21^Cip1/Waf1^, which suppress the activity of CDK4/6 and CDK2, respectively. This suppression keeps the Rb in a hypophosphorylated, active state, preventing E2F-driven transcription and S-phase entry. In senescence escape, downregulation or loss-of-function mutations in *CDKN2A* or *CDKN1A* genes can relieve this checkpoint, allowing CDK activity to resume. This leads to Rb hyperphosphorylation, E2F activation, and re-entry into the cell cycle, particularly in tumor cells under selective pressure or with accumulated genomic alterations [92]. Epigenetic deregulation is another major driver of senescence reversal. In stable senescence, SAHF help enforce cell cycle arrest by silencing proliferation-related genes via histone modifications such as H3K9me3 and H3K27me3. Enzymes like EZH2 (a methyltransferase within the PRC2 complex) maintain H3K27me3 marks on pro-growth genes. However, epigenetic plasticity, including loss of heterochromatin marks or upregulation of histone demethylases (e.g., KDM6A/UTX, KDM4), can destabilize these chromatin structures. This leads to derepression of E2F target genes, re-expression of cyclins, and reversal of the senescent phenotype [93, 94].

A critical facilitator of senescence escape is the AKT/mTOR signaling pathway, which regulates cell growth, survival, and metabolism. In senescent cells, mTORC1 activity is typically suppressed to conserve energy. However, reactivation of mTORC1—for instance, via PI3K activation, PTEN loss, or increased nutrient signaling—can promote biosynthesis and proliferation, undermining senescence maintenance. mTORC1 also suppresses autophagy, which is essential for sustaining the senescent state by removing damaged organelles and limiting ROS. Loss of autophagic flux in this context supports mitochondrial dysfunction reversal, increased ATP production, and cell cycle re-entry [6, 95, 96]. In parallel, senescence escape is tightly linked to mitochondrial dynamics and metabolic rewiring. While senescent cells exhibit increased mitochondrial mass and elevated ROS, escape is associated with mitochondrial clearance, reduced ROS levels, and a shift toward glycolytic metabolism (Warburg effect), facilitating renewed proliferation. This metabolic switch is often driven by c-Myc, HIF-1α, and PGC-1α, which reprogram cellular energy use and redox balance to a proliferative state [97, 98]. Importantly, immune evasion also contributes to the persistence and eventual escape of senescent tumor cells. When SASP signaling becomes chronic, it not only promotes inflammation and tissue remodeling but also attracts immunosuppressive cell types, including M2 macrophages and Tregs. These immune cells dampen cytotoxic responses, allowing senescent cells to persist and accumulate further mutations that facilitate escape. Moreover, senescent cells often upregulate PD-L1, allowing them to engage in immune checkpoint interactions and evade T cell-mediated clearance [99]. In some contexts, senescence escape leads to the emergence of stem-like, therapy-resistant tumor cells. These cells display markers of cancer stemness (e.g., ALDH1, CD44^high^/CD24^low^) and exhibit high tumor-initiating potential. They are thought to originate from a subpopulation of senescent cells that undergo transcriptional reprogramming through Wnt, Notch, or YAP/TAZ pathways. This escape-reprogramming phenomenon has been observed following genotoxic stress and is associated with increased tumor heterogeneity and metastatic potential [100–102].

This senescence reversal has been observed in cancer models following extended exposure to genotoxic stress or hypoxia and is associated with epigenetic deregulation and downregulation of CDK inhibitors [92]. These “escaped” cells not only regain proliferative capacity but may also exhibit stem-like features and enhanced metastatic potential. The tumor-promoting potential of senescence is further supported by studies in immune-suppressed or immune-excluded tumors, where the lack of proper immune clearance of senescent cells allows them to accumulate and exert deleterious effects. For instance, senescent fibroblasts within the TME can produce Fas ligand (FasL) and trigger apoptosis of infiltrating T and NK cells, thereby undermining anti-tumor immunity and aiding immune escape [103].

## Promising role of senolytics

Given the risk of senescence escape and the detrimental effects of lingering SASP, therapeutic approaches aimed at selectively eliminating senescent cells—senolytics—offer a rational complement to senescence-inducing therapies. Senolytics represent an emerging class of therapeutics that could significantly enhance the efficacy and safety of TIS (Table 3) [104]. While TIS serves as a potent anti-tumor mechanism by enforcing irreversible growth arrest and promoting immune activation through the SASP, the accumulation of senescent cells and chronic SASP can paradoxically promote inflammation, tumor progression, and immunosuppression if senescent cells are not efficiently cleared. This creates a therapeutic window for senolytics, which can be employed after the immune-priming phase of TIS to remove residual senescent cells, thereby mitigating the long-term pro-tumorigenic risks associated with chronic SASP [105–107]. For instance, a study showed that cancer therapies like doxorubicin and etoposide can induce tumor cell senescence, which may contribute to disease relapse. However, these TIS cells were found to be selectively sensitive to the senolytic agent ABT-263 (navitoclax). By disrupting BCL_XL_–BAX interactions, ABT-263 promotes apoptosis in senescent cells, effectively eliminating them. In vivo, sequential treatment with chemotherapy followed by ABT-263 led to sustained tumor suppression. These findings suggest that combining senescence-inducing therapies with senolytic agents may enhance treatment efficacy and reduce the risk of cancer recurrence [105]. Moreover, the timing and sequencing of senolytics are critical for maximizing synergy with immune therapies. Administering senolytics too early may prematurely deplete senescent cells before they can fully exert their immunostimulatory effects, such as SASP-mediated recruitment of immune effector cells. Conversely, delayed administration, after peak immune infiltration, allows for sufficient immune priming while minimizing chronic SASP-associated toxicity [108, 109]. This strategy is supported by preclinical models in which CDK4/6 inhibitors were used to induce senescence, followed by immune therapy and then senolytic treatment, resulting in improved tumor regression and reduced systemic inflammation [65]. Finally, senolytics may also target senescent stromal and endothelial cells within TME, which can support tumor survival and contribute to therapy resistance. Their clearance not only limits pro-tumorigenic signaling but may also enhance immune cell infiltration and drug delivery by normalizing the extracellular matrix and vasculature [110–112].

**Table 3:** Role of senolytics in optimizing therapy-induced senescence

Senolytic Agent/Class	Primary Target/Mechanism	Rationale in TIS Context	Preclinical/Clinical Evidence	Limitations/Challenges
Navitoclax (ABT-263)	BCL-2/BCL-xL inhibitor	Induces apoptosis in senescent cells by disrupting pro-survival BCL-2 family protein interactions	Shown to clear TIS cells after doxorubicin/etoposide; sequential use with chemotherapy reduces relapse in murine models	Thrombocytopenia due to BCL-xL inhibition; dosing and sequencing critical
ABT-737	BCL-2/BCL-xL/BCL-w inhibitor	Similar mechanism to navitoclax; triggers apoptosis in senescent cells dependent on anti-apoptotic BCL-2 proteins	Preclinical models demonstrate selective clearance of TIS cells	Limited oral bioavailability; off-target toxicities
Dasatinib (TKI)	SRC family kinases; multiple targets	Induces apoptosis in senescent fibroblasts and some tumor-associated senescent cells	Effective in combination with quercetin in senescent stromal clearance	Cell-type specific; less potent in epithelial senescent cells
Quercetin (flavonoid)	PI3K/AKT pathway, anti-oxidant activity	Promotes senescent cell death; enhances dasatinib effects (D + Q)	D + Q combination reduces senescent stromal burden in preclinical cancer and aging models	Limited bioavailability; pleiotropic effects
FOXO4-DRI peptide	Disrupts FOXO4–p53 interaction	Forces p53 nuclear exclusion, inducing apoptosis in senescent cells	Selective clearance of therapy-induced senescent cells in models; restores tissue function	Delivery and stability challenges; early-stage experimental
HSP90 inhibitors (e.g., 17-DMAG)	Inhibit heat shock protein 90, destabilizing survival signaling	Senescent cells are particularly dependent on HSP90 for proteostasis	Preclinical evidence supports senolytic activity against senescent tumor cells	Systemic toxicity limits clinical translation
Cardiac glycosides (e.g., digoxin, ouabain)	Inhibit Na+/K + ATPase, inducing apoptosis	Exploit metabolic vulnerability of senescent cells	Preclinical studies show selective elimination of senescent tumor cells	Narrow therapeutic index; cardiotoxicity risks
Fisetin (flavonoid)	Multiple signaling pathways (anti-oxidant, PI3K/AKT, NF-κB)	Promotes apoptosis and reduces SASP factors	Shown to clear senescent cells and reduce inflammation in models	Low potency compared to targeted senolytics
Emerging PROTAC-based senolytics	Targeted protein degradation of senescence survival factors	Potential for higher specificity and reduced off-target toxicity	Early preclinical development	Yet to be validated in cancer TIS context

## Combination of TIS and immunotherapies

Several rigorous preclinical studies show that conventional cytotoxics and targeted agents that induce senescence can sensitize tumors to immune checkpoint blockade [113]. In mouse models of breast and brain-metastatic breast cancer, induction of senescence with doxorubicin or combinations that trigger durable cell-cycle exit increased CD8 + T-cell infiltration and antigen-presentation signatures, and when combined with anti-PD-1/PD-L1 antibodies produced greater tumor regression and prolongation of survival than either therapy alone; depletion of CD8 + T cells abrogated the benefit, implicating adaptive immunity as the effector of synergy. These studies also report that SASP drives dendritic cell maturation and chemokine gradients that recruit effector T cells, providing a mechanistic link between TIS and increased ICI responsiveness [114]. Mechanistic molecular work has refined why senescent cells can both help and hinder immunotherapy. Recent reports demonstrate that senescent tumor cells often upregulate immunoregulatory molecules—most notably PD-L1—through transcriptional and post-translational mechanisms; one high-profile study identified ribophorin-1–dependent glycosylation as a regulator of PD-L1 stabilization in senescent cancer cells, which can paradoxically shield them from T-cell killing unless PD-1/PD-L1 is blocked. Thus, TIS can create a tumor state that is simultaneously more immunogenic (enhanced antigen presentation, SASP-driven recruitment) and more dependent on immune checkpoints for immune escape—explaining why combining TIS inducers with checkpoint blockade often yields additive or synergistic results in preclinical systems [115, 116]. CDK4/6 inhibitors are among the best-characterized clinically relevant senescence inducers with immunomodulatory effects. Multiple preclinical investigations reported that palbociclib/abemaciclib treatment increases Type I/II interferon signaling, MHC-I expression, and intratumoral T-cell activation; in murine tumor models these changes sensitize tumors to PD-1 blockade, producing improved tumor control versus monotherapy. Early-phase clinical work has explored this combination: a phase I/II trial combining palbociclib with pembrolizumab (and endocrine therapy where applicable) demonstrated that the regimen is feasible and signals of clinical activity exist in selected breast cancer cohorts, although definitive efficacy and optimal sequencing remain under study. Importantly, these clinical datasets emphasize dosing/sequence tradeoffs because CDK4/6 inhibitors also transiently suppress T-cell proliferation—an effect that can blunt combination benefit if timing is suboptimal [113, 117].

An emerging translational strategy is *induce-then-purge*: induce senescence to prime immune recognition and then eradicate residual senescent cells with senolytics (or by recruiting immune effectors) to prevent SASP-driven tumor relapse (Table 4). Preclinical models using BCL-2/BCL-xL inhibitors (e.g., navitoclax) demonstrate selective clearance of TIS cells, reduction of pro-tumorigenic SASP factors, and improved responses when senolytics are sequenced after senescence-inducing therapy. These data provide a rationale to combine senescence induction + ICI with either concurrent or sequential senolytic therapy to (a) maximize immune-mediated clearance and (b) avoid long-term SASP-mediated immune suppression or tumor promotion. Several early-phase clinical studies are evaluating navitoclax and other BCL-2 family inhibitors in cancer patients (safety/tolerability and PK are primary endpoints); translational arms of these trials are beginning to measure senescence and immune biomarkers to inform future combinatorial designs [118, 119]. Clinical evidence to date is encouraging but mixed and highlights critical variables that determine outcome: tumor type, the agent used to trigger senescence, timing/sequence relative to ICI, and the molecular profile of the SASP. Across reviews and translational reports, positive signals tend to cluster where senescence induction robustly increases antigen presentation and T-cell recruitment without prolonged SASP-mediated myeloid immunosuppression. Conversely, models and early clinical observations warn that chronic accumulation of senescent cells can induce immunosuppressive myeloid populations and upregulate immune checkpoints, potentially increasing immune-related adverse effects or limiting long-term benefit if senescent cells are not cleared. These insights motivate trial designs that incorporate serial immune and senescence biomarkers (e.g., p16/p21, SASP cytokines, MHC expression, PD-L1 glycosylation status) and test distinct sequencing strategies (concurrent vs. induction-then-ICI vs. induction-then-senolytic) [120, 121]. In sum, reported data support a strategy in which TIS is used to convert “cold” tumors into an immunologically inflamed state that is then exploited with immune checkpoint or adoptive therapies, while senolytics or immune clearance are deployed to remove persistent senescent cells and forestall SASP-driven relapse. The most compelling translational next steps—already underway in several trial consortia—are (1) biomarker-led patient selection, (2) carefully timed sequencing to preserve effector T-cell function, and (3) integrated correlative studies to define which senescent signatures predict durable responses versus those that predict immunosuppression or toxicity.

**Table 4:** Potential combination strategies of therapy-induced senescence, immunotherapies and senolytics

Strategy (short name)	Rationale	Timing/Sequencing	Candidate agents (examples)	Expected immune/TME effects	Biomarkers to monitor	Risks/mitigation
1. Concurrent TIS + ICI	Induce senescence to increase antigen presentation/SASP while simultaneously blocking immune checkpoints to enhance T-cell activity.	Overlap dosing of senescence-inducer and immune checkpoint inhibitor (concurrent). Short induction window preferred.	CDK4/6 inhibitor (palbociclib) + anti-PD-1/PD-L1 (pembrolizumab)	Rapid conversion of “cold” → “inflamed” TME; increased CD8⁺ infiltration, MHC-I upregulation	Tumor PD-L1, MHC-I, intratumoral CD8, IFN-γ signature, circulating SASP cytokines (IL-6, IL-8)	Risk: transient T-cell proliferation suppression from CDK4/6 — mitigate with dosing breaks or lower CDK4/6 exposure around ICI initiation.
2. Induce → Prime (ICI) → Purge (senolytic) (Induce–Prime–Purge)	Let senescence-established SASP recruit/activate immune cells, then remove residual senescent cells to prevent chronic SASP.	(1) TIS induction (days–weeks) → (2) peak immune priming (monitor) with ICI → (3) senolytic after evidence of immune infiltration (weeks after induction).	Inducer: doxorubicin or CDK4/6 inhibitor; ICI: anti-PD-1; senolytic: navitoclax or FOXO4-DRI	Optimal immune-mediated clearance, reduced chronic SASP and relapse risk	Kinetics of SASP (plasma IL-6/IL-8), intratumoral CD8/NK, senescence markers (p16/p21, SA-β-gal proxy), platelet counts (for navitoclax)	Timing critical—give senolytic only after immune priming to avoid removing antigenic/immune-activating senescent cells. Monitor hematologic toxicity.
3. TIS inducer → Senolytic (sequential, no ICI)	In tumors where immunotherapy is unsuitable, purge TIS cells after induction to avoid pro-tumorigenic chronic SASP.	Induce senescence (single/short course) → allow short interval (immune priming optional) → senolytic administration.	Genotoxic chemo (etoposide) → navitoclax or dasatinib + quercetin (D + Q)	Reduce residual pro-tumor SASP, limit metastasis-promoting effects	Tumor senescence load (p16/p21), circulating SASP; platelet/ECG monitoring	Risk of systemic toxicity; may blunt potential immune benefits if senolytic given too early.
4. TIS + ICI + Senolytic (triplet integrated)	Maximize antigenicity and effector function then remove senescent cells to limit chronic toxicity and escape clones.	Induction → short ICI window concurrent/overlap → senolytic after biomarker-confirmed immune activation (flexible sequencing)	CDK4/6 or AURKA inhibitor + anti-PD-1/CTLA-4 + navitoclax or PROTAC senolytic	Enhanced T-cell/NK killing, reduced SASP persistence and immunosuppressive myeloid recruitment	As above + myeloid markers (MDSC, M2 macrophages), PD-L1 glycosylation status	High toxicity risk (myelosuppression, immune AEs). Requires careful dose modification and biomarker-guided timing.
5. TIS + Adoptive cell therapy (ACT)	TIS increases antigen presentation and NKG2D ligand expression enhancing ACT recognition and activity.	Induce senescence → allow 3–10 day window for antigen upregulation → infuse CAR-T or TILs; consider senolytic later.	Inducer: AURKA inhibitor or radiation; ACT: CAR-T/TILs; senolytic: selective agent later	Improved ACT homing and killing; possible reduction of immune suppression when combined with senolytics	Tumor antigen/MHC expression, NKG2D ligands, ACT expansion/persistence, SASP kinetics	Risk: cytokine release with high SASP; mitigate with step-dose ACT and close monitoring.
6. Epigenetic modulator + TIS + ICI	Epigenetic drugs (EZH2/HDAC inhibitors) restore pro-inflammatory SASP in epigenetically repressed tumors and sensitize to TIS and ICI.	Epigenetic priming → TIS induction → combine with ICI; senolytic optionally after immune activation	EZH2 inhibitor + MEK/CDK4/6 inhibitor + anti-PD-1; follow with navitoclax	Reprogram SASP to immune-stimulatory profile; increased NK/T recruitment	Chromatin marks (H3K27me3), SASP gene expression, immune infiltration	Risk of enhanced toxicity and unpredictable SASP changes — require transcriptomic readouts.
7. Low-dose/Metronomic TIS induction + intermittent senolytic	Maintain periodic induction of senescence to keep TME inflamed while periodically clearing senescent cells to prevent accumulation.	Cyclic low-dose inducer (metronomic) with senolytic pulses between cycles	Low-dose anthracycline or CDK4/6 low-dose schedule + intermittent D + Q or PROTAC	Sustained immune activation with controlled SASP burden	Serial circulating SASP, immune activation markers, organ function	Complexity in scheduling; risk of cumulative toxicity — pilot biomarker trials needed.
8. Targeted TIS in stroma (senescent stroma purge + immunotherapy)	Many TMEs depend on senescent stromal cells; clearing stromal senescence may normalize vasculature and improve ICI delivery.	Induce stromal senescence (if necessary) or identify existing stromal senescence → senolytic → ICI	Senolytics preferential for stromal cells (D + Q, fisetin) + anti-PD-1	Decreased fibrosis, improved T-cell infiltration, better drug perfusion	ECM remodeling markers, vascular perfusion imaging, stromal senescence markers	Off-target loss of beneficial stromal cells; imaging-guided targeting may help.
9. TIS + oncolytic virus/in situ vaccination + senolytic	SASP enhances viral spread/immune priming; oncolytic viruses amplify antigen release and immune recruitment.	TIS induction → intratumoral oncolytic virus/vaccine → systemic ICI ± later senolytic	Radiation or chemo → T-VEC/TLR agonist → anti-PD-1 → senolytic	Potent local inflammation, dendritic cell maturation, systemic T-cell priming	Local cytokine surge, tumor viral load, DC activation markers	Risk of excess inflammation; control with staged dosing and anti-inflammatory rescue plans.

## Challenges and future directions

Despite its promise, leveraging TIS in cancer therapy presents several major challenges that must be addressed to optimize therapeutic outcomes. One of the most significant barriers is the heterogeneity and chronicity of the SASP. The composition of SASP varies not only between cell types and senescence inducers but also over time [76]. Acute SASP can be immunostimulatory, promoting immune cell recruitment and tumor clearance, whereas chronic SASP can lead to persistent inflammation, fibrosis, and the recruitment of immunosuppressive cell populations such as Tregs, M2-like macrophages, and MDSCs [122, 123]. This inflammatory and tumor-promoting shift in SASP composition can drive immune evasion, angiogenesis, and EMT, potentially facilitating tumor relapse or metastasis. Strategies to overcome SASP heterogeneity include targeted inhibition of specific SASP regulators, such as NF-κB, p38 MAPK, or IL-1α, and the use of epigenetic modulators like EZH2 inhibitors, which have been shown to reprogram SASP toward a more immunogenic profile [124, 125]. The successful clinical deployment of senescence-targeting therapies also hinges on the development of reliable biomarkers to monitor the induction, persistence, and clearance of senescent cells in patients. Unlike apoptosis, which has clear molecular hallmarks, senescence is defined by a complex and often context-dependent signature. Traditional markers such as senescence-associated (SA)-β-galactosidase activity, p16^INK4a^, p21^Cip1/Waf1^, and SAHF are informative but lack consistency across tissue types and treatment regimens. Moreover, they do not capture the dynamic nature of senescence or its immunological impact. Recent studies have identified SASP components, non-coding RNAs, and metabolic changes as potential biomarkers, and there is growing interest in circulating DNA, extracellular vesicles, and immunopeptidomics as minimally invasive tools to track senescence in real time [126–128]. Ultimately, a multi-parametric approach combining molecular, immunological, and metabolic signatures will be essential for personalized senescence-guided therapy [129, 130]. Overall, while TIS offers a compelling framework for enhancing cancer immunotherapy, addressing the challenges of SASP heterogeneity, immune targeting specificity, and biomarker development will be essential to realizing its full therapeutic potential. These future directions will require interdisciplinary collaboration across immunology, oncology, and systems biology to develop finely tuned, clinically translatable strategies.

## Conclusion

TIS represents a potent but double-edged mechanism in cancer therapy—capable of halting tumor progression and enhancing immune clearance, yet equally capable of driving chronic inflammation, immune suppression, and relapse if left unchecked. Major gaps remain in our ability to precisely modulate SASP, predict senescence dynamics in patients, and determine the optimal sequencing of senescence inducers, immunotherapies, and senolytics. Future efforts should prioritize the development of robust senescence biomarkers, the dissection of non-canonical and tissue-specific senescence pathways, and the integration of systems-level and longitudinal approaches to guide patient-specific interventions. Advancing these directions will be essential to transform TIS from a partly opportunistic phenomenon into a deliberately harnessed therapeutic axis for durable cancer control.

## Data Availability

No datasets were generated or analysed during the current study.

## References

[1] Prasanna PG, Citrin DE, Hildesheim J, Ahmed MM, Venkatachalam S, Riscuta G, Xi D, Zheng G, van Deursen J, Goronzy J, et al. Therapy-Induced senescence: opportunities to improve anticancer therapy. J Natl Cancer Inst. 2021;113:1285–98. 10.1093/jnci/djab064. 33792717 10.1093/jnci/djab064 PMC8486333

[2] Ewald JA, Desotelle JA, Wilding G, Jarrard DF. Therapy-Induced senescence in cancer. J Natl Cancer Inst. 2010;102:1536–46. 10.1093/jnci/djq364. 20858887 10.1093/jnci/djq364 PMC2957429

[3] Saleh T, Bloukh S, Carpenter VJ, Alwohoush E, Bakeer J, Darwish S, Azab B, Gewirtz DA. Therapy-Induced senescence: an old friend becomes the enemy. Cancers. 2020;12:822. 10.3390/cancers12040822. 32235364 10.3390/cancers12040822 PMC7226427

[4] Fitsiou E, Soto-Gamez A, Demaria M. Biological functions of Therapy-Induced senescence in cancer. Sem Cancer Biol. 2022;81:5–13. 10.1016/j.semcancer.2021.03.021. 10.1016/j.semcancer.2021.03.021 33775830

[5] Chibaya L, Snyder J, Ruscetti M. Senescence and the Tumor-Immune landscape: implications for cancer immunotherapy. Sem Cancer Biol. 2022;86:827–45. 10.1016/j.semcancer.2022.02.005. 10.1016/j.semcancer.2022.02.005 PMC9357237 35143990

[6] Liu Y, Lomeli I, Kron SJ. Therapy-Induced Cellular Senescence: Potentiating Tumor Elimination or Driving Cancer Resistance and Recurrence? Cells. 2024;13(15):1281. 10.3390/cells13151281. 10.3390/cells13151281 PMC11312217 39120312

[7] Kciuk M, Gielecińska A, Mujwar S, Kołat D, Kałuzińska-Kołat Ż, Celik I, Kontek R. Doxorubicin—An agent with multiple mechanisms of anticancer activity. Cells. 2023;12:659. 10.3390/cells12040659. 36831326 10.3390/cells12040659 PMC9954613

[8] Wei F, Hao P, Zhang X, Hu H, Jiang D, Yin A, Wen L, Zheng L, He JZ, Mei W, et al. Etoposide-Induced DNA damage affects multiple cellular pathways in addition to DNA damage response. Oncotarget. 2018;9:24122–39. 10.18632/oncotarget.24517. 29844877 10.18632/oncotarget.24517 PMC5963631

[9] Helt CE, Cliby WA, Keng PC, Bambara RA, O’Reilly MA. Ataxia telangiectasia mutated (ATM) and ATM and Rad3-Related protein exhibit selective target specificities in response to different forms of DNA damage. J Biol Chem. 2005;280:1186–92. 10.1074/jbc.M410873200. 15533933 10.1074/jbc.M410873200

[10] Maréchal A, Zou L. DNA damage sensing by the ATM and ATR kinases. Cold Spring Harb Perspect Biol. 2013;5:a012716. 10.1101/cshperspect.a012716. 24003211 10.1101/cshperspect.a012716 PMC3753707

[11] Fischer M. Census and evaluation of P53 target genes. Oncogene. 2017;36:3943–56. 10.1038/onc.2016.502. 28288132 10.1038/onc.2016.502 PMC5511239

[12] Hume S, Dianov GL, Ramadan K. A unified model for the G1/S cell cycle transition. Nucleic Acids Res. 2020;48:12483–501. 10.1093/nar/gkaa1002. 33166394 10.1093/nar/gkaa1002 PMC7736809

[13] Gong P, Guo Z, Wang S, Gao S, Cao Q. Histone phosphorylation in DNA damage response. Int J Mol Sci. 2025;26:2405. 10.3390/ijms26062405. 40141048 10.3390/ijms26062405 PMC11941871

[14] Xie A, Hartlerode A, Stucki M, Odate S, Puget N, Kwok A, Nagaraju G, Yan C, Alt FW, Chen J, et al. Distinct roles of Chromatin-Associated proteins MDC1 and 53BP1 in mammalian Double-Strand break repair. Mol Cell. 2007;28:1045–57. 10.1016/j.molcel.2007.12.005. 18158901 10.1016/j.molcel.2007.12.005 PMC2275782

[15] Rothkamm K, Barnard S, Moquet J, Ellender M, Rana Z, Burdak-Rothkamm S. DNA damage foci: meaning and significance. Environ Mol Mutagen. 2015;56:491–504. 10.1002/em.21944. 25773265 10.1002/em.21944

[16] Al-Khalaf HH, Mohideen P, Nallar SC, Kalvakolanu DV, Aboussekhra A. The Cyclin-Dependent kinase inhibitor p16INK4a physically interacts with transcription factor Sp1 and Cyclin-Dependent kinase 4 to transactivate MicroRNA-141 and MicroRNA-146b-5p spontaneously and in response to ultraviolet Light-Induced DNA damage. J Biol Chem. 2013;288:35511–25. 10.1074/jbc.M113.512640. 24163379 10.1074/jbc.M113.512640 PMC3853297

[17] Liu J-Y, Souroullas GP, Diekman BO, Krishnamurthy J, Hall BM, Sorrentino JA, Parker JS, Sessions GA, Gudkov AV, Sharpless NE. Cells Exhibiting Strong *P16*^*INK4a*^ Promoter Activation in Vivo Display Features of Senescence. Proc. Natl. Acad. Sci. U.S.A. 2019;116(7):2603–2611. 10.1073/pnas.1818313116. 10.1073/pnas.1818313116 PMC6377452 30683717

[18] Salotti J, Johnson PF. Regulation of senescence and the SASP by the transcription factor C/EBPβ. Exp Gerontol. 2019;128:110752. 10.1016/j.exger.2019.110752. 31648009 10.1016/j.exger.2019.110752

[19] Cuollo L, Antonangeli F, Santoni A, Soriani A. The Senescence-Associated secretory phenotype (SASP) in the challenging future of cancer therapy and Age-Related diseases. Biology. 2020;9:485. 10.3390/biology9120485. 33371508 10.3390/biology9120485 PMC7767554

[20] Robert M, Kennedy BK, Crasta KC. Therapy-Induced senescence through the redox lens. Redox Biol. 2024;74:103228. 10.1016/j.redox.2024.103228. 38865902 10.1016/j.redox.2024.103228 PMC11215421

[21] Li D, Yu Q, Wu R, Tuo Z, Wang J, Ye L, Shao F, Chaipanichkul P, Yoo KH, Wei W, et al. Interactions between oxidative stress and senescence in cancer: Mechanisms, therapeutic Implications, and future perspectives. Redox Biol. 2024;73:103208. 10.1016/j.redox.2024.103208. 38851002 10.1016/j.redox.2024.103208 PMC11201350

[22] Oliveira BL. Biophysical systems approach to identifying the pathways of acute and chronic doxorubicin mitochondrial cardiotoxicity. PLoS Comput Biol. 2016;12:e1005214. 10.1371/journal.pcbi.1005214. NiedererS. A. 27870850 10.1371/journal.pcbi.1005214 PMC5117565

[23] Kursunluoglu G, Kayali HA, Taskiran D. The effect of cisplatin toxicity and capsaicin on electron transport chain in liver and kidney of Sprague Dawley rats. Cell Biochem Biophys. 2014;69:707–16. 10.1007/s12013-014-9857-z. 24648159 10.1007/s12013-014-9857-z

[24] Srinivas US, Tan BWQ, Vellayappan BA, Jeyasekharan AD. ROS and the DNA damage response in cancer. Redox Biol. 2018;25:101084. 10.1016/j.redox.2018.101084. 30612957 10.1016/j.redox.2018.101084 PMC6859528

[25] Shi T, van Soest DMK, Polderman PE, Burgering BMT, Dansen TB. DNA damage and oxidant stress activate P53 through differential upstream signaling pathways. Free Radic Biol Med. 2021;172:298–311. 10.1016/j.freeradbiomed.2021.06.013. 34144191 10.1016/j.freeradbiomed.2021.06.013

[26] Borodkina A, Shatrova A, Abushik P, Nikolsky N, Burova E. Interaction between ROS dependent DNA Damage, mitochondria and P38 MAPK underlies senescence of human adult stem cells. Aging. 2014;6:481–95. 24934860 10.18632/aging.100673 PMC4100810

[27] Canovas B, Nebreda AR. Diversity and versatility of P38 kinase signalling in health and disease. Nat Rev Mol Cell Biol. 2021;22:346–66. 10.1038/s41580-020-00322-w. 33504982 10.1038/s41580-020-00322-w PMC7838852

[28] Smolková K, Mikó E, Kovács T, Leguina-Ruzzi A, Sipos A, Bai P. Nuclear factor erythroid 2-Related factor 2 in regulating cancer metabolism. Antioxid Redox Signal. 2020;33:966–97. 10.1089/ars.2020.8024. 31989830 10.1089/ars.2020.8024 PMC7533893

[29] Baird L, Taguchi K, Zhang A, Takahashi Y, Suzuki T, Kensler TW, Yamamoto M. A NRF2-Induced secretory phenotype activates immune surveillance to remove irreparably damaged cells. Redox Biol. 2023;66:102845. 10.1016/j.redox.2023.102845. 37597423 10.1016/j.redox.2023.102845 PMC10458321

[30] Gall Trošelj K, Tomljanović M, Jaganjac M, Matijević Glavan T, Čipak Gašparović A, Milković L, Borović Šunjić S, Buttari B, Profumo E, Saha S, et al. Oxidative stress and cancer heterogeneity orchestrate NRF2 roles relevant for therapy response. Molecules. 2022;27:1468. 10.3390/molecules27051468. 35268568 10.3390/molecules27051468 PMC8912061

[31] Nelson G, Kucheryavenko O, Wordsworth J, von Zglinicki T. The senescent bystander effect is caused by ROS-Activated NF-κB signalling. Mech Ageing Dev. 2018;170:30–6. 10.1016/j.mad.2017.08.005. 28837845 10.1016/j.mad.2017.08.005 PMC5861994

[32] Lingappan K. NF-κB in oxidative stress. Curr Opin Toxicol. 2018;7:81–6. 10.1016/j.cotox.2017.11.002. 29862377 10.1016/j.cotox.2017.11.002 PMC5978768

[33] Barnes RP, Fouquerel E, Opresko PL. The impact of oxidative DNA damage and stress on telomere homeostasis. Mech Ageing Dev. 2019;177:37–45. 10.1016/j.mad.2018.03.013. 29604323 10.1016/j.mad.2018.03.013 PMC6162185

[34] von Zglinicki T. Oxidative stress and cell senescence as drivers of ageing: chicken and egg. Ageing Res Rev. 2024;102:102558. 10.1016/j.arr.2024.102558. 39454760 10.1016/j.arr.2024.102558

[35] Wagner V, Gil J. Senescence as a therapeutically relevant response to CDK4/6 inhibitors. Oncogene. 2020;39:5165–76. 10.1038/s41388-020-1354-9. 32541838 10.1038/s41388-020-1354-9 PMC7610384

[36] Wang X, Zhao S, Xin Q, Zhang Y, Wang K, Li M. Recent progress of CDK4/6 inhibitors’ current practice in breast cancer. Cancer Gene Ther. 2024;31:1283–91. 10.1038/s41417-024-00747-x. 38409585 10.1038/s41417-024-00747-x PMC11405274

[37] Grant GD, Cook JG. The Temporal regulation of S phase proteins during G1. Adv Exp Med Biol. 2017;1042:335–69. 10.1007/978-981-10-6955-0_16. 29357066 10.1007/978-981-10-6955-0_16 PMC5909198

[38] Al Bitar S, Gali-Muhtasib H. The role of the Cyclin dependent kinase inhibitor P21cip1/Waf1 in targeting cancer: molecular mechanisms and novel therapeutics. Cancers (Basel). 2019;11:1475. 10.3390/cancers11101475. 31575057 10.3390/cancers11101475 PMC6826572

[39] Wang L, Lankhorst L, Bernards R. Exploiting senescence for the treatment of cancer. Nat Rev Cancer. 2022;22:340–55. 10.1038/s41568-022-00450-9. 35241831 10.1038/s41568-022-00450-9

[40] Liu Y, Deng Y, Yang C, Naranmandura H. Double-Faced immunological effects of CDK4/6 inhibitors on cancer treatment: challenges and perspectives. Bioeng (Basel). 2024;11:1084. 10.3390/bioengineering11111084. 10.3390/bioengineering11111084 PMC11591775 39593745

[41] Castilho RM, Castilho LS, Palomares BH, Squarize CH. Determinants of chromatin organization in aging and Cancer—Emerging opportunities for epigenetic therapies and AI technology. Genes. 2024;15:710. 10.3390/genes15060710. 38927646 10.3390/genes15060710 PMC11202709

[42] Lee DH, Imran M, Choi JH, Park YJ, Kim YH, Min S, Park TJ, Choi YW. CDK4/6 inhibitors induce breast cancer senescence with enhanced Anti-tumor Immunogenic properties compared with DNA‐damaging agents. Mol Oncol. 2023;18:216–32. 10.1002/1878-0261.13541. 37854019 10.1002/1878-0261.13541 PMC10766199

[43] Charles A, Bourne CM, Korontsvit T, Aretz ZEH, Mun SS, Dao T, Klatt MG, Scheinberg DA. Low-Dose CDK4/6 inhibitors induce presentation of pathway specific MHC ligands as potential targets for cancer immunotherapy. Oncoimmunology. 2021;10:1916243. 10.1080/2162402X.2021.1916243. 34104540 10.1080/2162402X.2021.1916243 PMC8158036

[44] Zhang S, Xu Q, Sun W, Zhou J, Zhou J. Immunomodulatory effects of CDK4/6 inhibitors. Biochim Et Biophys Acta (BBA) - Reviews Cancer. 2023;1878(4):188912. 10.1016/j.bbcan.2023.188912. 10.1016/j.bbcan.2023.188912 37182667

[45] Liu C, Huang Y, Cui Y, Zhou J, Qin X, Zhang L, Li X, Li Y, Guo E, Yang B, et al. The immunological role of CDK4/6 and potential mechanism exploration in ovarian cancer. Front Immunol. 2022;12. 10.3389/fimmu.2021.799171. 10.3389/fimmu.2021.799171 PMC8795791 35095879

[46] Du R, Huang C, Liu K, Li X, Dong Z. Targeting AURKA in cancer: molecular mechanisms and opportunities for cancer therapy. Mol Cancer. 2021;20. 10.1186/s12943-020-01305-3. 10.1186/s12943-020-01305-3 PMC7809767 33451333

[47] Caputo E, Miceli R, Motti ML, Taté R, Fratangelo F, Botti G, Mozzillo N, Carriero MV, Cavalcanti E, Palmieri G, et al. AurkA inhibitors enhance the effects of B-RAF and MEK inhibitors in melanoma treatment. J Transl Med. 2014;12:216. 10.1186/s12967-014-0216-z. 25074438 10.1186/s12967-014-0216-z PMC4237855

[48] Vilgelm AE, Johnson CA, Prasad N, Yang J, Chen S-C, Ayers GD, Pawlikowski JS, Raman D, Sosman JA, Kelley M, et al. Connecting the dots: Therapy-Induced senescence and a Tumor-Suppressive immune microenvironment. J Natl Cancer Inst. 2016;108. 10.1093/jnci/djv406. 10.1093/jnci/djv406 PMC4849355 26719346

[49] Vilgelm A, Richmond A. Combined therapies that induce senescence and stabilize P53 block melanoma growth and prompt antitumor immune responses. Oncoimmunology. 2015;4:e1009299. 10.1080/2162402X.2015.1009299. 26405565 10.1080/2162402X.2015.1009299 PMC4570092

[50] Pradhan T, Gupta O, Singh G, Monga V. Aurora kinase inhibitors as potential anticancer agents: recent advances. Eur J Med Chem. 2021;221:113495. 10.1016/j.ejmech.2021.113495. 34020340 10.1016/j.ejmech.2021.113495

[51] Ma HT, Poon RYC. Aurora kinases and DNA damage response. Mutat Res. 2020;821:111716. 10.1016/j.mrfmmm.2020.111716. 32738522 10.1016/j.mrfmmm.2020.111716

[52] Vora S, Andrew A, Kumar RP, Nazareth D, Bonfim-Melo A, Lim Y, Ong XY, Fernando M, He Y, Hooper JD, et al. Aurora B inhibitors promote RB hypophosphorylation and senescence independent of P53-Dependent CDK2/4 Inhibition. Cell Death Dis. 2024;15:1–9. 10.1038/s41419-024-07204-5. 39521795 10.1038/s41419-024-07204-5 PMC11550316

[53] Kreuger IZM, Slieker RC, van Groningen T, van Doorn R. Therapeutic strategies for targeting *CDKN2A* loss in melanoma. J Invest Dermatology. 2023;143(e1):18–25. 10.1016/j.jid.2022.07.016. 10.1016/j.jid.2022.07.016 36123181

[54] Liu Y, Hawkins OE, Su Y, Vilgelm AE, Sobolik T, Thu Y, Kantrow S, Splittgerber RC, Short S, Amiri KI, et al. Targeting Aurora kinases limits tumour growth through DNA Damage-mediated senescence and Blockade of NF‐κB impairs this Drug‐induced senescence. EMBO Mol Med. 2013;5:149–66. 10.1002/emmm.201201378. 23180582 10.1002/emmm.201201378 PMC3569660

[55] Kobayashi H, Azumi M, Hayashi S, Sato K, Aoki N, Kimura S, Kakizaki H, Nagato T, Harabuchi Y, Tateno M, et al. Characterization of human CD4 helper T cell responses against Aurora kinase A. Cancer Immunol Immunother. 2010;59:1029–39. 10.1007/s00262-010-0826-0. 20182874 10.1007/s00262-010-0826-0 PMC11030889

[56] Sagiv A, Burton DGA, Moshayev Z, Vadai E, Wensveen F, Ben-Dor S, Golani O, Polic B, Krizhanovsky V. NKG2D ligands mediate immunosurveillance of senescent cells. Aging. 2016;8:328–44. 26878797 10.18632/aging.100897 PMC4789586

[57] Meng B, Zhao X, Jiang S, Xu Z, Li S, Wang X, Ma W, Li L, Liu D, Zheng J, et al. AURKA Inhibitor-Induced PD-L1 upregulation impairs antitumor immune responses. Front Immunol. 2023;14. 10.3389/fimmu.2023.1182601. 10.3389/fimmu.2023.1182601 PMC10536236 37781397

[58] Ghosh S, O’Hara MP, Sinha P, Mazumdar T, Yapindi L, Sastry JK, Johnson FM. Targeted Inhibition of Aurora kinase A promotes immune checkpoint Inhibition efficacy in human Papillomavirus-Driven cancers. J Immunother Cancer. 2025;13:e009316. 10.1136/jitc-2024-009316. 39773561 10.1136/jitc-2024-009316 PMC11749607

[59] Chandler H, Peters G. Stressing the cell cycle in senescence and aging. Curr Opin Cell Biol. 2013;25:765–71. 10.1016/j.ceb.2013.07.005. 23916530 10.1016/j.ceb.2013.07.005

[60] Fiorentino FP, Symonds CE, Macaluso M, Giordano A. Senescence and P130/Rbl2: A new beginning to the end. Cell Res. 2009;19:1044–51. 10.1038/cr.2009.96. 19668264 10.1038/cr.2009.96

[61] Freund A, Patil CK, Campisi J. p38MAPK is a novel DNA damage Response-Independent regulator of the Senescence-Associated secretory phenotype. EMBO J. 2011;30:1536–48. 10.1038/emboj.2011.69. 21399611 10.1038/emboj.2011.69 PMC3102277

[62] Roy S, Roy S, Rana A, Akhter Y, Hande MP, Banerjee B. The role of P38 MAPK pathway in P53 compromised state and telomere mediated DNA damage response. Mutat Research/Genetic Toxicol Environ Mutagen. 2018;836:89–97. 10.1016/j.mrgentox.2018.05.018. 10.1016/j.mrgentox.2018.05.018 30389168

[63] Hydbring P, Castell A, Larsson L-G. MYC modulation around the CDK2/P27/SKP2 axis. Genes. 2017;8:174. 10.3390/genes8070174. 28665315 10.3390/genes8070174 PMC5541307

[64] Zimmerli D, Brambillasca CS, Talens F, Bhin J, Linstra R, Romanens L, Bhattacharya A, Joosten SEP, Da Silva AM, Padrao N, et al. MYC promotes Immune-Suppression in Triple-Negative breast cancer via Inhibition of interferon signaling. Nat Commun. 2022;13:6579. 10.1038/s41467-022-34000-6. 36323660 10.1038/s41467-022-34000-6 PMC9630413

[65] Ruscetti M. Abstract SY24-03: modulating cellular senescence to reinstate natural killer cell immunity for pancreatic cancer immunotherapy. Cancer Res. 2022;82(SY24–03–SY24–03). 10.1158/1538-7445.AM2022-SY24-03.

[66] Paluvai H, Di Giorgio E, Brancolini C. The histone code of senescence. Cells. 2020;9:466. 10.3390/cells9020466. 32085582 10.3390/cells9020466 PMC7072776

[67] Vora S, Andrew A, Kumar RP, Nazareth D, Fernando M, Jones MJ, He Y, Hooper JD, McMillan NA, Urosevic J et al. Aurora B Inhibition promotes a Hyper-Polyploid state and continued endomitotic cycles in RB and P53 defective cells 2024, 2024.03.27.585450.

[68] Chicas A, Wang X, Zhang C, McCurrach M, Zhao Z, Mert O, Dickins RA, Narita M, Zhang M, Lowe SW. Dissecting the unique role of the retinoblastoma tumor suppressor during cellular senescence. Cancer Cell. 2010;17:376–87. 10.1016/j.ccr.2010.01.023. 20385362 10.1016/j.ccr.2010.01.023 PMC2889489

[69] Sagiv A, Krizhanovsky V. Immunosurveillance of senescent cells: the bright side of the senescence program. Biogerontology. 2013;14:617–28. 10.1007/s10522-013-9473-0. 24114507 10.1007/s10522-013-9473-0

[70] Fetarayani D, Kahdina M, Waitupu A, Pratiwi L, Ningtyas MC, Adytia GJ, Sutanto H. Immunosenescence and the geriatric giants: molecular insights into aging and healthspan. Med Sci. 2025;13:100. 10.3390/medsci13030100. 10.3390/medsci13030100 PMC12372083 40843723

[71] Zhao H, Wu L, Yan G, Chen Y, Zhou M, Wu Y, Li Y. Inflammation and tumor progression: signaling pathways and targeted intervention. Signal Transduct Target Ther. 2021;6:263. 10.1038/s41392-021-00658-5. 34248142 10.1038/s41392-021-00658-5 PMC8273155

[72] Ruscetti M, Morris JP, Mezzadra R, Russell J, Leibold J, Romesser PB, Simon J, Kulick A, Ho Y, Fennell M, et al. Senescence-Induced vascular remodeling creates therapeutic vulnerabilities in pancreas cancer. Cell. 2020;181:424–e44121. 10.1016/j.cell.2020.03.008. 32234521 10.1016/j.cell.2020.03.008 PMC7278897

[73] Hartupee C, Nagalo BM, Chabu CY, Tesfay MZ, Coleman-Barnett J, West JT, Moaven O. Pancreatic cancer tumor microenvironment is a major therapeutic barrier and target. Front Immunol. 2024;15. 10.3389/fimmu.2024.1287459. 10.3389/fimmu.2024.1287459 PMC10867137 38361931

[74] Schmiedel D, Mandelboim O. NKG2D Ligands–Critical targets for cancer immune escape and therapy. Front Immunol. 2018;9:2040. 10.3389/fimmu.2018.02040. 30254634 10.3389/fimmu.2018.02040 PMC6141707

[75] Muñoz DP, Yannone SM, Daemen A, Sun Y, Vakar-Lopez F, Kawahara M, Freund AM, Rodier F, Wu JD, Desprez P-Y, et al. Targetable mechanisms driving immunoevasion of persistent senescent cells link Chemotherapy-Resistant cancer to aging. JCI Insight. 2019;4:e124716. 10.1172/jci.insight.124716. 10.1172/jci.insight.124716 PMC6675550 31184599

[76] Sutanto H, Fetarayani D, Narendra MR, Nasution SA. The role of the Senescence-Associated secretory phenotype in cardiovascular disease among the elderly. Eur J Intern Med. 2025;0. 10.1016/j.ejim.2025.106488. 10.1016/j.ejim.2025.106488 40858450

[77] Prieto LI, Baker DJ. Cellular senescence and the immune system in cancer. Gerontology. 2019;65:505–12. 10.1159/000500683. 31212284 10.1159/000500683 PMC6703936

[78] Davalos AR, Coppe J-P, Campisi J, Desprez P-Y. Senescent cells as a source of inflammatory factors for tumor progression. Cancer Metastasis Rev. 2010;29:273–83. 10.1007/s10555-010-9220-9. 20390322 10.1007/s10555-010-9220-9 PMC2865636

[79] Carpenter VJ, Saleh T, Gewirtz DA. Senolytics for cancer therapy: is all that glitters really gold? Cancers (Basel). 2021;13:723. 10.3390/cancers13040723. 33578753 10.3390/cancers13040723 PMC7916462

[80] Fan H, Qiao Z, Li J, Shang G, Shang C, Chen S, Leng Z, Su H, Kou H, Liu H. Recent advances in Senescence-Associated secretory phenotype and osteoporosis. Heliyon. 2024;10:e25538. 10.1016/j.heliyon.2024.e25538. 38375248 10.1016/j.heliyon.2024.e25538 PMC10875379

[81] Blackford AN, Jackson SP, ATM, ATR. The trinity at the heart of the DNA damage response. Mol Cell. 2017;66:801–17. 10.1016/j.molcel.2017.05.015. 28622525 10.1016/j.molcel.2017.05.015

[82] Wang W, Mani AM, Wu Z-H. DNA Damage-Induced nuclear Factor-Kappa B activation and its roles in cancer progression. J Cancer Metastasis Treat. 2017;3:45–59. 10.20517/2394-4722.2017.03. 28626800 10.20517/2394-4722.2017.03 PMC5472228

[83] Huang B, Lang X, Li X. The role of IL-6/JAK2/STAT3 signaling pathway in cancers. Front Oncol. 2022;12:1023177. 10.3389/fonc.2022.1023177. 36591515 10.3389/fonc.2022.1023177 PMC9800921

[84] Takasugi M, Yoshida Y, Hara E, Ohtani N. The role of cellular senescence and SASP in tumour microenvironment. FEBS J. 2023;290:1348–61. 10.1111/febs.16381. 35106956 10.1111/febs.16381

[85] Wang Y, Li T, Wang F, Yao X, Bai Q, Su H, Liu J, Wang L, Tan R. The dual role of cellular senescence in macrophages: unveiling the hidden driver of Age-Related inflammation in kidney disease. Int J Biol Sci. 2025;21:632–57. 10.7150/ijbs.104404. 39781471 10.7150/ijbs.104404 PMC11705649

[86] Salminen A, Kaarniranta K, Kauppinen A, Immunosenescence. The potential role of Myeloid-Derived suppressor cells (MDSC) in Age-Related immune deficiency. Cell Mol Life Sci. 2019;76:1901–18. 10.1007/s00018-019-03048-x. 30788516 10.1007/s00018-019-03048-x PMC6478639

[87] Zhou L, Murphy KC, Snyder J, DeMarco KD, Ma B, Ruscetti M. Abstract B035: leveraging Therapy-Induced senescence for prostate cancer immunotherapy. Cancer Res. 2023;83:B035–035. 10.1158/1538-7445.PRCA2023-B035.

[88] Coppé J-P, Desprez P-Y, Krtolica A, Campisi J. The Senescence-Associated secretory phenotype: the dark side of tumor suppression. Annu Rev Pathol. 2010;5:99–118. 10.1146/annurev-pathol-121808-102144. 20078217 10.1146/annurev-pathol-121808-102144 PMC4166495

[89] Vilgelm AE, Johnson CA, Prasad N, Yang J, Chen S-C, Ayers GD, Sosman JA, Ecsedy JA, Yu S, Levy SE, et al. Abstract 5126: the link between therapy-Induced senescence and Anti-Tumor immune microenvironment in melanoma. Cancer Res. 2016;76:5126–5126. 10.1158/1538-7445.AM2016-5126.

[90] Evangelou K, Belogiannis K, Papaspyropoulos A, Petty R, Gorgoulis VG. Escape from senescence: molecular basis and therapeutic ramifications. J Pathol. 2023;260:649–65. 10.1002/path.6164. 37550877 10.1002/path.6164

[91] Saleh T. Therapy-Induced senescence is finally Escapable, what is next? Cell Cycle. 2024;23:713–21. 10.1080/15384101.2024.2364579. 38879812 10.1080/15384101.2024.2364579 PMC11229739

[92] Nair J, Muley P, Dutt S. Therapy induced senescence and its implications in cancer. Biomed Res J. 2017;4. 10.4103/2349-3666.240590.

[93] Cheng Y, He C, Wang M, Ma X, Mo F, Yang S, Han J, Wei X. Targeting epigenetic regulators for cancer therapy: mechanisms and advances in clinical trials. Sig Transduct Target Ther. 2019;4:1–39. 10.1038/s41392-019-0095-0. 10.1038/s41392-019-0095-0 PMC6915746 31871779

[94] Flavahan WA, Gaskell E, Bernstein BE. Epigenetic plasticity and the hallmarks of cancer. Science. 2017;357:eaal2380. 10.1126/science.aal2380. 28729483 10.1126/science.aal2380 PMC5940341

[95] Astle MV, Hannan KM, Ng PY, Lee RS, George AJ, Hsu AK, Haupt Y, Hannan RD, Pearson RB. AKT induces senescence in human cells via mTORC1 and P53 in the absence of DNA damage: implications for targeting mTOR during malignancy. Oncogene. 2012;31:1949–62. 10.1038/onc.2011.394. 21909130 10.1038/onc.2011.394 PMC3325598

[96] Fakhri S, Zachariah Moradi S, DeLiberto LK, Bishayee A. Cellular senescence signaling in cancer: A novel therapeutic target to combat human malignancies. Biochem Pharmacol. 2022;199:114989. 10.1016/j.bcp.2022.114989. 35288153 10.1016/j.bcp.2022.114989

[97] Li M, Durbin KR, Sweet SMM, Tipton JD, Zheng Y, Kelleher NL. Oncogene induced cellular senescence elicits an Anti-Warburg effect. Proteomics. 2013;13. 10.1002/pmic.201200298. 10.1002/pmic.201200298 PMC3867201 23798001

[98] Zhang X, Gao Y, Zhang S, Wang Y, Du Y, Hao S, Ni T. The regulation of cellular senescence in cancer. Biomolecules. 2025;15:448. 10.3390/biom15030448. 40149983 10.3390/biom15030448 PMC11940315

[99] Hu H, Wang Q, Yu D, Tao X, Guo M, Tian S, Zhang Q, Xu M, Geng X, Zhang H, et al. Berberine derivative B68 promotes tumor immune clearance by Dual-Targeting BMI1 for senescence induction and CSN5 for PD‐L1 degradation. Adv Sci. 2025;12:2413122. 10.1002/advs.202413122. 10.1002/advs.202413122 PMC11831439 39721027

[100] Vipparthi K, Hari K, Chakraborty P, Ghosh S, Patel AK, Ghosh A, Biswas NK, Sharan R, Arun P, Jolly MK et al. Emergence of Hybrid States of Stem-like Cancer Cells Correlates with Poor Prognosis in Oral Cancer. *iScience* 2022, *25*, 104317. 10.1016/j.isci.2022.104317 10.1016/j.isci.2022.104317 PMC9114525 35602941

[101] Loh J-J, Ma S. Hallmarks of cancer stemness. Cell Stem Cell. 2024;31:617–39. 10.1016/j.stem.2024.04.004. 38701757 10.1016/j.stem.2024.04.004

[102] Santinon G, Brian I, Pocaterra A, Romani P, Franzolin E, Rampazzo C, Bicciato S, Dupont S. dNTP metabolism links mechanical cues and YAP/TAZ to cell growth and Oncogene-Induced senescence. EMBO J. 2018;37:e97780. 10.15252/embj.201797780. 29650681 10.15252/embj.201797780 PMC5983219

[103] Cruz M, Dulong J, Sonn A, Moquin-Beaudry G, Benabdallah B, Le O, Beauséjour C. Senescent Fibroblasts Support Tumor Growth by Inducing Immune Cell Death in Humanized Models 2024.

[104] Chaib S, Tchkonia T, Kirkland JL. Cellular senescence and senolytics: the path to the clinic. Nat Med. 2022;28:1556–68. 10.1038/s41591-022-01923-y. 35953721 10.1038/s41591-022-01923-y PMC9599677

[105] Saleh T, Carpenter VJ, Tyutyunyk-Massey L, Murray G, Leverson JD, Souers AJ, Alotaibi MR, Faber AC, Reed J, Harada H, et al. Clearance of Therapy-Induced senescent tumor cells by the senolytic ABT-263 via interference with BCL-XL -BAX interaction. Mol Oncol. 2020;14:2504–19. 10.1002/1878-0261.12761. 32652830 10.1002/1878-0261.12761 PMC7530780

[106] Czajkowski K, Herbet M, Murias M, Piątkowska-Chmiel I, Senolytics. Charting a new course or enhancing existing Anti-Tumor therapies? Cell Oncol. 2025;48:351–71. 10.1007/s13402-024-01018-5. 10.1007/s13402-024-01018-5 PMC11996976 39633108

[107] Wang Z, Gao J, Xu C. Targeting metabolism to influence cellular senescence a promising Anti-Cancer therapeutic strategy. Biomed Pharmacother. 2024;177:116962. 10.1016/j.biopha.2024.116962. 38936195 10.1016/j.biopha.2024.116962

[108] Hickson LJ, Langhi Prata LGP, Bobart SA, Evans TK, Giorgadze N, Hashmi SK, Herrmann SM, Jensen MD, Jia Q, Jordan KL, et al. Senolytics decrease senescent cells in humans: preliminary report from a clinical trial of dasatinib plus Quercetin in individuals with diabetic kidney disease. EBioMedicine. 2019;47:446–56. 10.1016/j.ebiom.2019.08.069. 31542391 10.1016/j.ebiom.2019.08.069 PMC6796530

[109] Short S, Fielder E, Miwa S, von Zglinicki T. Senolytics and Senostatics as Adjuvant Tumour Therapy. *EBioMedicine* 2019, *41*, 683–692. 10.1016/j.ebiom.2019.01.056 10.1016/j.ebiom.2019.01.056 PMC6441870 30737084

[110] Jiang B, Zhang W, Zhang X, Sun Y. Targeting senescent cells to reshape the tumor microenvironment and improve anticancer efficacy. Sem Cancer Biol. 2024;101:58–73. 10.1016/j.semcancer.2024.05.002. 10.1016/j.semcancer.2024.05.002 38810814

[111] Takasugi M, Yoshida Y, Ohtani N. Cellular senescence and the tumour microenvironment. Mol Oncol. 2022;16:3333–51. 10.1002/1878-0261.13268. 35674109 10.1002/1878-0261.13268 PMC9490140

[112] D’Ambrosio M, Gil J. Reshaping of the tumor microenvironment by cellular senescence: an opportunity for senotherapies. Dev Cell. 2023;58:1007–21. 10.1016/j.devcel.2023.05.010. 37339603 10.1016/j.devcel.2023.05.010

[113] Deng J, Wang ES, Jenkins RW, Li S, Dries R, Yates K, Chhabra S, Huang W, Liu H, Aref AR, et al. CDK4/6 Inhibition augments antitumor immunity by enhancing T-Cell activation. Cancer Discov. 2018;8:216–33. 10.1158/2159-8290.CD-17-0915. 29101163 10.1158/2159-8290.CD-17-0915 PMC5809273

[114] Uceda-Castro R, Margarido AS, Cornet L, Vegna S, Hahn K, Song J-Y, Putavet DA, van Geldorp M, Çitirikkaya CH, de Keizer PLJ, et al. Re-Purposing the pro-Senescence properties of doxorubicin to introduce immunotherapy in breast cancer brain metastasis. Cell Rep Med. 2022;3:100821. 10.1016/j.xcrm.2022.100821. 36384097 10.1016/j.xcrm.2022.100821 PMC9729880

[115] Hwang HJ, Kang D, Shin J, Jung J, Ko S, Jung KH, Hong S-S, Park JE, Oh MJ, An HJ, et al. Therapy-Induced senescent cancer cells contribute to cancer progression by promoting ribophorin 1-Dependent PD-L1 upregulation. Nat Commun. 2025;16. 10.1038/s41467-024-54132-1. 10.1038/s41467-024-54132-1 PMC11699195 39753537

[116] Oesterreich S, Aird KM. Senescence and immunotherapy: redundant Immunomodulatory pathways promote resistance. Cancer Immunol Res. 2023;11:401–4. 10.1158/2326-6066.CIR-23-0051. 36826438 10.1158/2326-6066.CIR-23-0051 PMC11221415

[117] Yuan Y, Lee J, Yost SE, Frankel PH, Ruel C, Egelston CA, Guo W, Padam S, Tang A, Martinez N, et al. Phase I/II trial of Palbociclib, Pembrolizumab, and letrozole in patients with hormone receptor positive metastatic breast cancer. Eur J Cancer. 2021;154:11–20. 10.1016/j.ejca.2021.05.035. 34217908 10.1016/j.ejca.2021.05.035 PMC8691850

[118] Zhu Y, Tchkonia T, Fuhrmann-Stroissnigg H, Dai HM, Ling YY, Stout MB, Pirtskhalava T, Giorgadze N, Johnson KO, Giles CB, et al. Identification of a novel senolytic Agent, Navitoclax, targeting the Bcl‐2 family of Anti‐apoptotic factors. Aging Cell. 2016;15:428–35. 10.1111/acel.12445. 26711051 10.1111/acel.12445 PMC4854923

[119] AbbVie A. *Phase 1 Open-Label Study Evaluating the Safety and Tolerability, and Pharmacokinetics of Navitoclax Monotherapy and in Combination With Ruxolitinib in Myeloproliferative Neoplasm Subjects*; clinicaltrials.gov, 2025.

[120] Saleh T, Greenberg EF, Faber AC, Harada H, Gewirtz DA. A critical appraisal of the utility of targeting Therapy-Induced senescence for cancer treatment. Cancer Res. 2025;85:1755–68. 10.1158/0008-5472.CAN-24-2219. 40036150 10.1158/0008-5472.CAN-24-2219

[121] Luo J, Sun T, Liu Z, Liu Y, Liu J, Wang S, Shi X, Zhou H. Persistent accumulation of Therapy-Induced senescent cells: an obstacle to Long-Term cancer treatment efficacy. Int J Oral Sci. 2025;17:59. 10.1038/s41368-025-00380-w. 40750580 10.1038/s41368-025-00380-w PMC12317027

[122] Bitencourt TC, Vargas JE, Silva AO, Fraga LR, Filippi-Chiela E. Subcellular Structure, Heterogeneity, and plasticity of senescent cells. Aging Cell. 2024;23:e14154. 10.1111/acel.14154. 38553952 10.1111/acel.14154 PMC11019148

[123] Kirschner K, Rattanavirotkul N, Quince MF, Chandra T. Functional heterogeneity in senescence. Biochem Soc Trans. 2020;48:765–73. 10.1042/BST20190109. 32369550 10.1042/BST20190109 PMC7329341

[124] Chibaya L, Murphy KC, DeMarco KD, Gopalan S, Liu H, Parikh CN, Lopez-Diaz Y, Faulkner M, Li J, Morris JP, et al. EZH2 Inhibition remodels the inflammatory Senescence-Associated secretory phenotype to potentiate pancreatic cancer immune surveillance. Nat Cancer. 2023;4:872–92. 10.1038/s43018-023-00553-8. 37142692 10.1038/s43018-023-00553-8 PMC10516132

[125] Dong Z, Luo Y, Yuan Z, Tian Y, Jin T, Xu F. Cellular senescence and SASP in tumor progression and therapeutic opportunities. Mol Cancer. 2024;23:181. 10.1186/s12943-024-02096-7. 39217404 10.1186/s12943-024-02096-7 PMC11365203

[126] Domen A, Deben C, Verswyvel J, Flieswasser T, Prenen H, Peeters M, Lardon F, Wouters A. Cellular senescence in cancer: clinical detection and prognostic implications. J Exp Clin Cancer Res. 2022;41:360. 10.1186/s13046-022-02555-3. 36575462 10.1186/s13046-022-02555-3 PMC9793681

[127] Pacifico F, Magni F, Leonardi A, Crescenzi E. Therapy-Induced senescence: novel approaches for markers identification. IJMS. 2024;25:8448. 10.3390/ijms25158448. 39126015 10.3390/ijms25158448 PMC11313450

[128] Itahana K, Campisi J, Dimri GP. Methods to detect biomarkers of cellular senescence: the Senescence-Associated Beta-Galactosidase assay. Methods Mol Biol. 2007;371:21–31. 10.1007/978-1-59745-361-5_3. 17634571 10.1007/978-1-59745-361-5_3

[129] Doolittle ML, Saul D, Kaur J, Rowsey JL, Vos SJ, Pavelko KD, Farr JN, Monroe DG, Khosla S. Multiparametric senescent cell phenotyping reveals targets of senolytic therapy in the aged murine skeleton. Nat Commun. 2023;14:4587. 10.1038/s41467-023-40393-9. 37524694 10.1038/s41467-023-40393-9 PMC10390564

[130] Mansfield L, Ramponi V, Gupta K, Stevenson T, Mathew AB, Barinda AJ, Herbstein F, Morsli S. Emerging insights in senescence: pathways from preclinical models to therapeutic innovations. Npj Aging. 2024;10:1–33. 10.1038/s41514-024-00181-1. 39578455 10.1038/s41514-024-00181-1 PMC11584693

